# Why Do Muse Stem Cells Present an Enduring Stress Capacity? Hints from a Comparative Proteome Analysis

**DOI:** 10.3390/ijms22042064

**Published:** 2021-02-19

**Authors:** Mustafa B. Acar, Domenico Aprile, Serife Ayaz-Guner, Huseyin Guner, Coskun Tez, Giovanni Di Bernardo, Gianfranco Peluso, Servet Ozcan, Umberto Galderisi

**Affiliations:** 1 Graduate School of Natural and Applied Sciences, Erciyes University, Kayseri 38039, Turkey; acarmburak@gmail.com; 2 Genome and Stem Cell Center (GENKOK), Erciyes University, Kayseri 38039, Turkey; tezc@erciyes.edu.tr; 3 Department of Experimental Medicine, Luigi Vanvitelli Campania University, 80138 Naples, Italy; domenico.aprile@unicampania.it (D.A.); gianni.dibernardo@unicampania.it (G.D.B.); 4 Department of Molecular Biology and Genetics, Faculty of Life and Natural Science, Abdullah Gul University, Kayseri 38090, Turkey; serife.ayaz@agu.edu.tr (S.A.-G.); huseyin.guner@agu.edu.tr (H.G.); 5 Department of Biology, Faculty of Sciences, Erciyes University, Kayseri 38039, Turkey; 6 Sbarro Institute for Cancer Research and Molecular Medicine, Center for Biotechnology, Temple University, Philadelphia, PA 19122, USA; 7 Research Institute on Ecosystems (IRET), CNR, 80131 Naples, Italy; gianfranco.peluso@cnr.it

**Keywords:** stem cells, DNA damage, oxidative stress, mesenchymal stromal cells

## Abstract

Muse cells are adult stem cells that are present in the stroma of several organs and possess an enduring capacity to cope with endogenous and exogenous genotoxic stress. In cell therapy, the peculiar biological properties of Muse cells render them a possible natural alternative to mesenchymal stromal cells (MSCs) or to in vitro-generated pluripotent stem cells (iPSCs). Indeed, some studies have proved that Muse cells can survive in adverse microenvironments, such as those present in damaged/injured tissues. We performed an evaluation of Muse cells’ proteome under basic conditions and followed oxidative stress treatment in order to identify ontologies, pathways, and networks that can be related to their enduring stress capacity. We executed the same analysis on iPSCs and MSCs, as a comparison. The Muse cells are enriched in several ontologies and pathways, such as endosomal vacuolar trafficking related to stress response, ubiquitin and proteasome degradation, and reactive oxygen scavenging. In Muse cells, the protein–protein interacting network has two key nodes with a high connectivity degree and betweenness: NFKB and CRKL. The protein NFKB is an almost-ubiquitous transcription factor related to many biological processes and can also have a role in protecting cells from apoptosis during exposure to a variety of stressors. CRKL is an adaptor protein and constitutes an integral part of the stress-activated protein kinase (SAPK) pathway. The identified pathways and networks are all involved in the quality control of cell components and may explain the stress resistance of Muse cells.

## 1. Introduction

All cells of an organism receive several intrinsic and extrinsic stresses on a daily basis. The cell metabolism, and the nucleic acids’ replication and transcription are the main intrinsic stress events, as they are related to production of reactive oxygen species (ROS) that damage proteins, lipids, DNA, and RNA. The extrinsic stressors are environmental, chemical, and physical genotoxic agents that may negatively affect cellular activities. Cells have a “quality control” program that promote their functional recovery following stress. This program relies on correct DNA repair to completely recover the performance of damaged cells and also systems, ensuring that degraded/misfolded proteins do not accumulate within cells [1,2]. The quality control must be fully active in long-lived stem cells, as these cells must retain functionality to support tissue homeostasis, in spite of several rounds of intrinsic and extrinsic stresses.

The mesenchymal stromal cells (MSCs), present in the stroma of almost every tissue, are a heterogenous population composed of distinct subtypes: Stem cells, progenitors, and differentiated cells. Several findings have evidenced that stem cells of MSCs can produce a mesodermal progeny of osteocytes, chondrocytes, adipocytes, and muscle cells [3]. Dezawa et al. identified and isolated an SSEA-3-positive stem cell population within MSCs that they named multilineage-differentiating, stress-enduring (Muse) cells, given their stress tolerance. These cells, representing around 1–3% of MSCs, are self-renewable and are able to differentiate into endodermal, ectodermal, and mesodermal phenotypes [4,5]. Further studies demonstrated that endogenous Muse cells contribute to tissue repair at serious damage sites given their stress resistance that allows them to survive in hostile microenvironments, such as those present in damaged tissues [6].

In our previous findings, we demonstrated that Muse stem cells have a higher DNA repair capacity compared to other cells [7,8]. In the current study, we aimed to evaluate what the other aspects of the cell quality control program are that permit Muse cells to have an enduring stress resistance. We analyzed the proteome of Muse cells in basic conditions, followed oxidative stress, and made a comparison with the proteome of MSCs and the non-Muse cell population (SSEA-3-negative fraction of MSCs). In addition, we compared the Muse cell proteome with induced pluripotent stem cells (iPSCs), which represent an artificial pluripotent stem cell population obtainable by in vitro reprogramming of adult somatic cells. The iPSCs are of interest as they show high similarity with embryonic stem cells (ESCs) and can produce mesodermal, endodermal, and ectodermal derivatives. Nowadays, iPSCs are used as in vitro models for the studies of many diseases, and there are also clinical trials based on iPSCs for cell therapy [9,10].

## 2. Results

**Figure 1:**
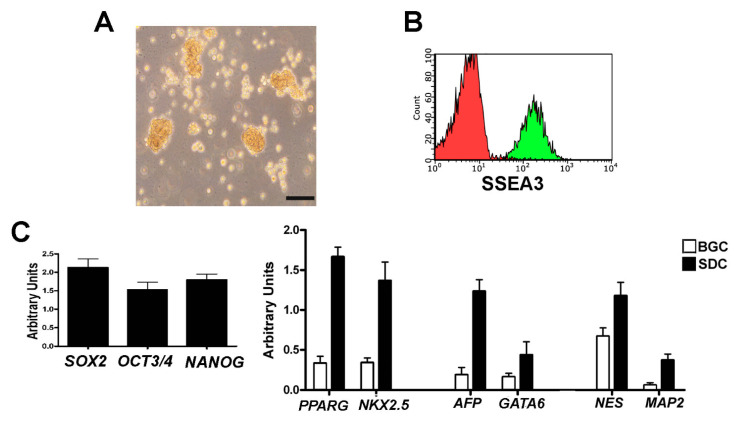
In vitro characterization of Muse cells. Panel (**A**) The picture shows a representative image of Muse cells grown in suspension and visualized through an inverted microscope (Leica DMIL 090-135.001). The black bar corresponds to 100 µM. Panel (**B**) After isolation, Muse cells were grown for 7–10 days and then the SSEA-3 expression was evaluated with flow cytometry analysis. The histograms show SSEA-3-positive cells (green) and the flow cytometry control reaction (red). Panel (**C**) RT-PCR analysis of potency (left graph) and differentiation markers (right graph) expressed by Muse cells grown either in basal growing conditions, as floating spheres, or in spontaneous differentiating conditions on culture dishes treated with 0.1% gelatin. The graph shows mRNA expression levels of genes of interest that were normalized to GAPDH mRNA level, which was selected as an internal control. The data are expressed as arbitrary units with standard deviation. BGC: Basal growth condition; SDC: Spontaneous differentiation condition. NES: NESTIN; AFP: Alpha-fetoprotein.

Under our experimental conditions, the MSCs obtained from American Type Culture Collection (ATCC) expressed the specific markers for defining multipotent mesenchymal stromal cells. These MSCs were CD90, CD73, CD105-positive, CD45, and CD34-negative [11] (Supplementary file 1). We then demonstrated that our isolation and cultivation method produced Muse cells as reported by Kuroda and co-workers [12]. Flow cytometry analysis showed that SSEA-3-positive Muse cells, which we isolated from MSCs, continued to express the SSEA-3 marker during in vitro cultivation (Figure 1A,B), while non-Muse cells did not express this antigen throughout the cultivation period. We evaluated by RT-PCR (Reverse Transcriptase-Polymerase Chain Reaction) the expression of potency markers such as NANOG, OCT3/4, and SOX2 on Muse cells grown in suspension (Figure 1C). Then, we evaluated the commitment of Muse cells into mesodermal, endodermal, and ectodermal lineages with the spontaneous differentiation assay. We observed the expression of GATA6 and AFP (endodermal lineage), NESTIN and MAP2 (ectodermal lineage), and NKX2-5 and PPARG (mesodermal lineage) by RT-PCR (Figure 1C). Non-Muse cells did not express either potency markers in basal growth conditions or lineage commitment markers after treatment for spontaneous differentiation (data not shown).

We verified that iPSCs, which we obtained from fibroblasts transfected with episomal reprograming plasmid, possessed the key features of pluripotent stem cells. Following transfection, putative iPSCs were grown for 12 days on feeder cells and were then isolated for characterization. The iPSCs expressed alkaline phosphatase; moreover, they were OCT3/4, SOX2, TRA-1-60, and SSEA-4-positive (Supplementary file 2).

### 2.1. LC-MS/MS Protein Analysis 

**Figure 2:**
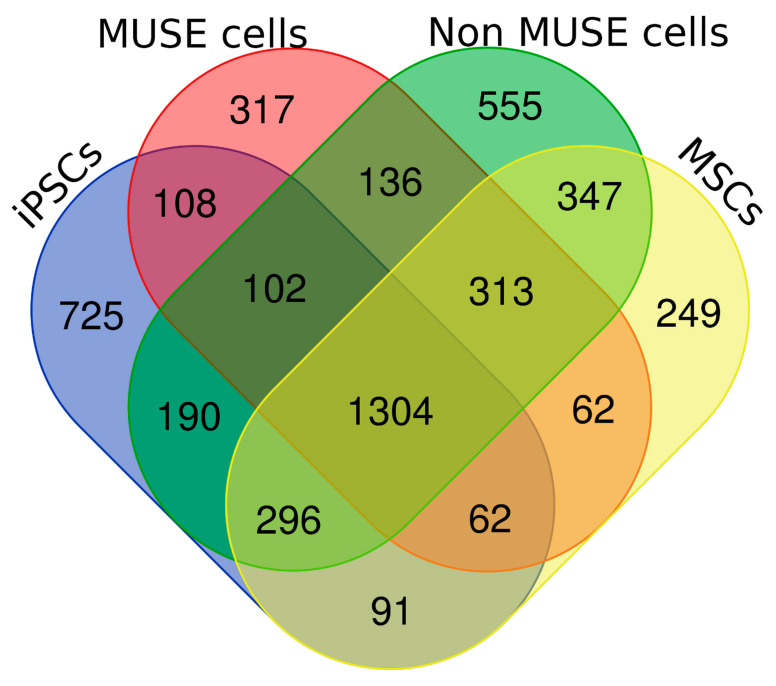
Venn diagram analysis. Venn diagram showing common and specific proteins among cell lysates obtained from in vitro-generated pluripotent stem cells (iPSCs), mesenchymal stromal cells (MSCs), Muse cells, and non-Muse cells. For every experimental condition, we performed biological and technical replicates. The picture refers to proteins that were consistently present in biological and technical replicates.

We performed LC-MS/MS analyses of peptides from the tryptic digestion of cell lysate samples obtained from iPSCs, MSCs, Muse cells, and non-Muse cells. Each sample had two biological replicates. We identified 2878 proteins in iPSCs, 2513 in MSCs, 2569 in Muse cells, and 3035 in non-Muse cells. The Venn analysis showed that, besides a core of 1304 proteins present in all cell samples, each cell type contained specific proteins (Figure 2 and Supplementary file 3). 

### 2.2. Gene Ontology Analysis of Cell Proteome Samples 

Differences in protein content among cell types prompted us to dissect protein distinctiveness by means of bioinformatics tools. Ontology terms are sets of proteins with relations between them. We performed a gene ontology (GO) analysis on our samples to identify the overrepresented ontology terms in our datasets compared to a reference human protein set. GO analysis was performed on a reduced set of GO terms to avoid redundancy in protein classification. We selected the ontological terms according to the following GO domains: Cellular components, protein classes, molecular functions, biological processes, and pathways. The proteome content of each cell type was classified in dozens of ontologies (Supplementary file 4). We then executed a Venn diagram analysis to combine the data of all experimental conditions in order to find both the specific and the common ontologies among the proteomes of iPSCs, MSCs, Muse cells, and non-Muse cells (Supplementary file 5). We identified 44 iPSC-specific ontologies (GO biological process) and, within this class, eight were associated with RNA metabolism, five with DNA replication/turnover, and three with chromatin organization (Table 1). Several terms out of the 28 specific GO terms in Muse cells belong to vesicle trafficking, response to endoplasmic stress, and quality control of cellular components (ubiquitin process, regulation of proteolysis, and cellular response to topologically incorrect proteins) (Table 1).

The MSCs’ proteome was also enriched in proteins involved in vesicle trafficking, while non-Muse cells exhibited sets of proteins associated with the cellular response to chemokines (Table 1). The analysis of cell-type-specific GO molecular function ontologies further evidenced that the iPSC proteome was enriched in protein classes associated with DNA/RNA anabolic and catabolic processes (Table 2). GO molecular function ontologies that were specifically present in the proteome of Muse cells additionally indicated that Muse cells were enriched in proteins involved in the quality control of cell macromolecules and also in response to oxidative stress (Table 2).

The analysis of cell-type-specific GO pathways (Table 2) and of GO cellular components (Supplementary file 5) further supported the observation that the main ontologies in iPSCs were associated with nucleic acid metabolism, while in Muse cells, they were coupled with “quality control” processes. In addition, GO pathways analysis evidenced that iPSCs, Muse cells, and MSCs were enriched in ontologies related to energy metabolism (Table 2). Non-Muse cell ontologies were associated with ion cell transmembrane transport and VEGF signaling (Table 2).

### 2.3. Pathway Analysis

Broad classification of proteome content by GO analysis gave us a general view of the most significant protein groups (ontologies) present in the experimental datasets. We then aimed to rank proteins according to their significance for the biological processes and molecular functions we identified with GO. To this end, the key assumption is that the reactivity of a protein found in a given ontology reflects its biological importance for the processes and functions associated with that ontology. We performed Reactome analysis of our protein datasets. In this bioinformatics scrutiny, a “reaction” is any event (binding, activation, translocation, degradation, etc.) that modifies the state of a biological molecule [13,14]. In this context, the most important proteins of a given ontology are part of the same Reactome pathway. Reactome analysis identified 104 pathways in iPSCs, while in MSCs, Muse cells, and non-Muse cells, the pathway numbers were 96, 93, and 87, respectively (Supplementary file 6). We then performed Venn analysis to determine the cell-type-specific pathways (Table 3). 

Of interest, in iPSCs, the overrepresented pathways belong to DNA duplication and cell division. Other pathways are associated with DNA repair. These Reactome results further confirm that “cell machinery” in iPSCs has a strong bias toward cell duplication and nucleic acid metabolism. The overrepresented pathways exclusively present in Muse cells are related to ontologies we identified with PANTHER analysis, such as the endosomal/vacuolar pathway leading to antigen processing and the post-chaperonin tubulin folding pathway (Table 3). The pathways found in MSCs and non-Muse cells cannot be directly associated with the ontology groups we identified with PANTHER GO evaluation (Table 3).

### 2.4. Protein–Protein Network Analysis

**Figure 3:**
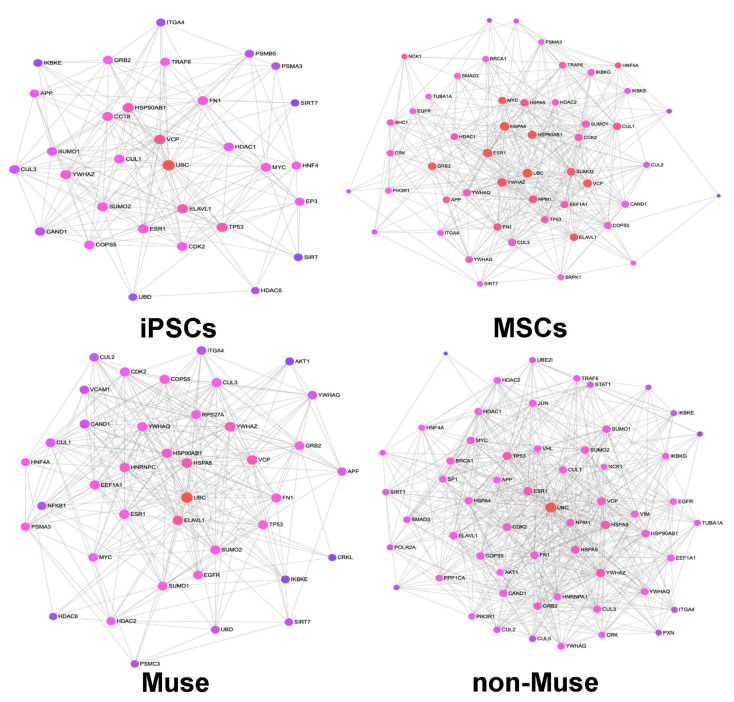
Minimum IMEx Interactome Networks. The picture shows the IMEx Networks generated with nodes having a connectivity degree higher than 100 and a betweenness higher than 10,000. For each node, the size is based on its degree values, with a big size for large degree values. The color switching from violet to red is proportional to betweenness centrality values, with red indicating the highest values.

The network construction allows a clear resolution of the biological context of the analyzed proteins. In this way, relevant biological pathways and molecules that interact with the protein list of interest are linked into a “functional whole.” Mapping the experimental protein datasets to a selected underlying database generates networks. Then, a search algorithm identifies proteins that directly interact with proteins of the experimental dataset, referred to as seeds. A large protein list (>1000 proteins) may produce complex networks. We reduced complexity by generating networks with Minimum IMEx Interactome Network, which trims the networks to keep only seeds and their connecting nodes [15]. Node degree and betweenness centrality are important properties of a network. The node degree indicates the number of edges that connect a node, while betweenness indicates how central a node is in a given network. Within the generated networks, we selected only nodes having a high node degree (>100) and betweenness (>10,000) for the construction of a “core network” that allows an effective data visualization and interpretation (Supplementary file 7). The iPSCs, MSCs, Muse cells, and non-Muse cells, in spite of their differences in biological properties and phenotypes, have a core network with a common group of key nodes that are associated with basic cellular functions, such as cell proliferation, differentiation, apoptosis (TP53, CMYC), and ubiquitin-related protein turnover (COPS5, CAND1, CUL1, CUL3, UBC, UBD) (Figure 3 and Table 4).

Besides these common nodes, every cell type has specific nodes belonging to the core network. For example, NFKB and CRKL are present in the network of Muse cells (Figure 3, Table 4). NFKB is an almost ubiquitous transcription factor present in almost all cell types. It is the endpoint of many signal transduction pathways that are initiated by diverse stimuli and are related to many biological processes (inflammation, immunity, differentiation, tumorigenesis, and apoptosis). In detail, NFKB has a key role protecting the cells from apoptosis during exposure to a variety of stressors [16,17].

CRKL is an adaptor protein that can sense diverse signals and can produce several biological responses. In particular, CRKL may promote cell proliferation and apoptosis resistance and constitutes an integral part of the stress-activated protein kinase (SAPK) pathway [18,19,20].

The EP300 and CCT8 are two key nodes present in the iPSCs’ core network. Of interest, EP300 is a histone acetyltransferase that plays a fundamental role in regulating stemness and pluripotency in embryonic stem cells [21]. The specific key nodes present in MSCs are mostly pleiotropic signal transduction proteins (MAPK1, SRPK1, SHC1). In non-Muse cells, several specific key nodes are proteins acting at nuclear levels (transcription factors, modulator of DNA replication) (Figure 3).

### 2.5. In Search of Muse Cells’ “Golden Bullet”

Our research approach was based on a top-down strategy, which started from the identification of global regulatory modules (ontologies, pathways, networks) that may be related to the peculiar biological properties of Muse cells. Then, the analysis focused on the 317 proteins we found only in the Muse cell dataset that may be part of the identified regulatory modules. To this end, we manually inspected the proteins present in the Muse cell proteome (Figure 2). We found five proteins that may play a major role in the stress resistance capacity of Muse cells: SELONOM, SELENOBP, OXR1, GPX8, and FANCM. Among these proteins, SELONOM, OXR1, and GPX8 have well-characterized antioxidant activity to protect cells against oxidative stress [22,23,24,25].

SELENOBP1 plays important roles in protein degradation, intra-Golgi transport, cell, redox modulation, and the metabolism of sulfur-containing molecules [26]. FANCM is part of the Fanconi anemia (FA) core complex, which contributes to the functionality of DNA repair machinery [27].

### 2.6. Genotoxic Stress and Its Consequences for Cell Functions

**Figure 4:**
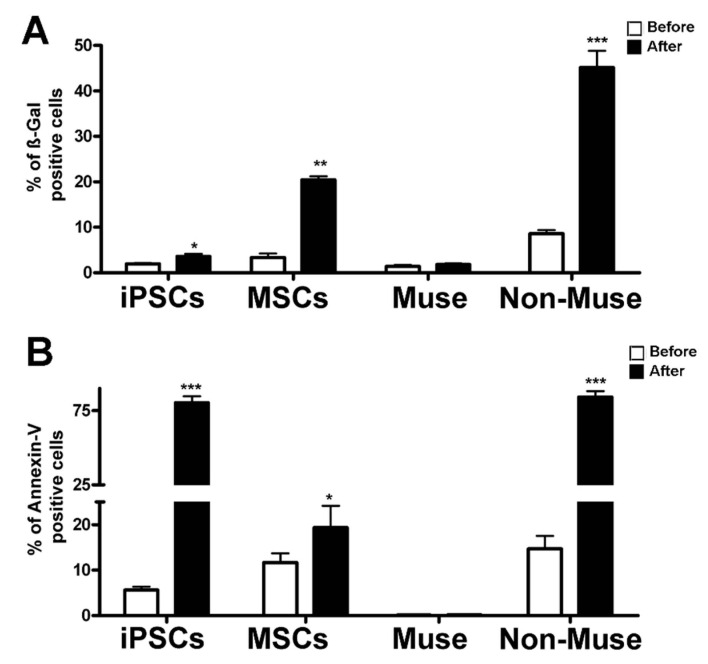
Apoptosis and senescence assays. Panel (**A**) The graph shows the mean percentage value of senescent cells determined by ß-galactosidase assay. For each cell type, the difference in senescence level was evaluated before and 48 h after H_2_O_2_ treatment. Data are expressed with SD (*n* = 3 for each experimental condition), * *p* < 0.05, ** *p* < 0.01, *** *p* < 0.001. Panel (**B**) The histogram shows the mean percentage of Annexin V-positive cells. For each cell type, the difference in senescence level was determined before and 24 h after H_2_O_2_ treatment. Data are expressed with SD (*n* = 3 for each experimental condition), * *p* < 0.05, *** *p* < 0.001.

We previously demonstrated that Muse cells have an efficient DNA damage sensing and repair capacity [7]. In this context, we evaluated changes in the proteome composition of iPSCs, MSCs, Muse cells, and non-Muse cells 12, 24, and 48 h following an oxidative stress (H_2_O_2_ treatment). In parallel, we determined apoptosis and senescence, as possible final endpoints of stress treatment. The apoptosis level was evaluated 12 h post-stress, while senescence was determined 48 h following H_2_O_2_ treatment. The rationale of this choice resided in the consideration that, following genotoxic injury, apoptosis is an event occurring before senescence. As we already demonstrated, Muse cells cope with DNA injury better than non-Muse cells and MSCs, as they did not show a significant increase in apoptosis and senescence, while MSCs and, to a greater extent, non-Muse cells showed an increment in both phenomena (Figure 4). The H_2_O_2_ treatment promoted massive apoptosis in iPSCs rather than senescence (Figure 4).

Having demonstrated that the genotoxic insult produced negative outputs mainly in iPSCs, MSCs, and non-Muse cells, we analyzed differences in proteome content between Muse cells and the other cell types that may justify the distinctness of Muse cells.

### 2.7. Changes in Proteome Profiles Following Genotoxic Stress

**Figure 5:**
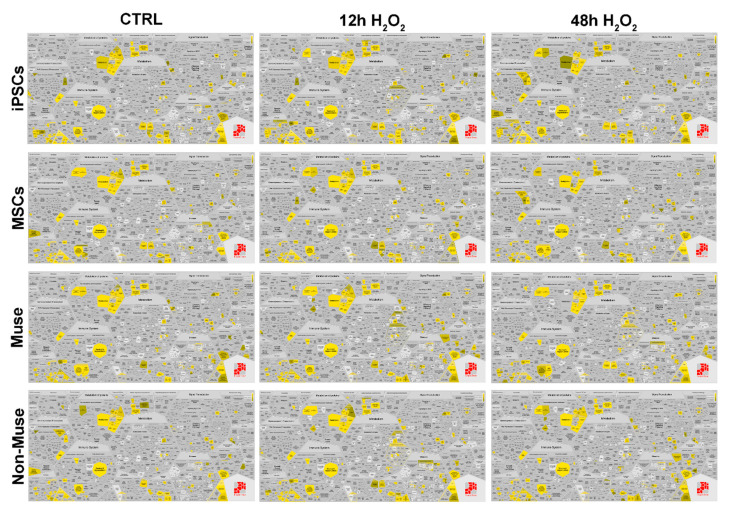
Reactome analysis of overrepresented pathways. Reactome analysis of protein datasets obtained from iPSCs, MSCs, Muse cells, and non-Muse cells before and after H_2_O_2_ treatment. Data are shown by Voronoi tessellation, which gives a general pathways overview. The overrepresented pathways are depicted in yellow (*p* < 0.05).

We compared the protein contents of the different cell types before and after H_2_O_2_ treatment by Reactome analysis of protein datasets obtained with LC-MS/MS (Supplementary file 3, 6). We visualized data by Voronoi tessellation, which gives a general pathways overview (Figure 5 and Supplementary file 8). 

**Figure 6:**
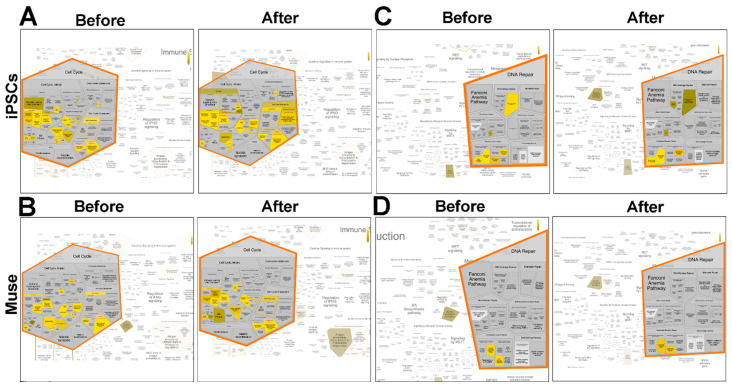
Details of Reactome analysis by Voronoi tessellation (**A**–**D**). Reactome analysis of protein datasets obtained from iPSCs and Muse cells before and 24 h after H_2_O_2_ treatment. Data shown are of Voronoi tessellation in detail. The overrepresented pathways (*p* < 0.05) are depicted in yellow.

In the Voronoi visualization, for a given pathway, represented as a cell, there is a corresponding region consisting of all other pathways closer to that one than to any other. The Voronoi visualization allowed a quick identification of the overrepresented pathway in each experimental condition (see yellow and green cells in Figure 5 and Figure 6). 

An in-depth look into Reactome analysis showed that following genotoxic stress, some biological processes (e.g., cell cycle; DNA repair; vacuolar trafficking, ubiquitination, and proteasome degradation; signal transduction) showed significant changes in the associated pathways.

Following DNA damage, iPSCs temporarily (12 and 24 h post-stress) upregulated the signal from unattached kinetochores to delay anaphase. This event was accompanied at 48 h by the silencing of pathways that promote cell cycle progression through the degradation by the ubiquitin pathway of P27 and P21, two cyclin kinase inhibitors (CKIs). This last event occurred earlier in Muse cells 24 h following stress and was associated with the activation of pathways associated with the G2 phase checkpoint (Figure 6A,B). In non-Muse cells and MSCs, both the silencing of CKI degradation and signals from unattached kinetochores did not occur.

An interesting difference between iPSCs and all the other cell types is that iPSCs activate the DNA damage bypass following stress injury (Figure 6C,D). This latter process is also referred to as translesion DNA synthesis (TLS), which results in nucleotide misincorporation opposite DNA lesions. This process allows cell survival in spite of the accumulation of DNA mutations [28].

As we underlined in previous paragraphs, Muse cells have an active system of vacuolar trafficking associated with antigen presentation, elimination of damaged proteins by ubiquitination, and phagosome degradation. This complex system of “quality control” is not affected by DNA damage events, rather, its pathways are further activated 48 h post-stress. These events occur only in Muse cells and not in the other cell types (Figure 5). Analysis of signal transduction showed that the MAP2K/MAPK pathway was always active in Muse cells, either in basal conditions and after stress induction, while in other cell types, it was switched off or transiently activated (Figure 5 and Supplementary file 8).

## 3. Discussion

LC-MS/MS protein identification and subsequent gene ontology, pathway, and network evaluations allowed us to perform an unbiased and hypothesis-free-manner analysis of Muse cells’ proteome to establish the basis for dissecting molecular events associated with their stress-enduring capacity.

The literature analysis of proteome studies showed that methods for protein cell extraction, digestion, and fractionation before MS/MS may profoundly affect the final outputs. Moreover, proteins coming from cell lysates present a wide dynamic range of concentrations. This causes the most abundant signal to mask the signal of low-abundance proteins (cytokines, growth factors, transcription factors, etc.) [29]. These phenomena can create discrepancies among published data on the proteome content of a given cell type in a specific experimental condition. In this context, we analyzed the proteome content of Muse cells, in basic conditions and after oxidative stress, and compared it with the proteomes of iPSCs and MSCs, analyzed with our methods, to have a homogenous reference group. This approach granted us a better comparison among the different cell types; nevertheless, the intrinsic limit of our LC/MS-MS analysis allowed us to identify only a fraction of the whole proteome content for every experimental condition. Further fine-tuning studies will be important to identify subsets of proteins we overlooked in the current investigation.

The integrity of the proteome is essential for cell viability and all cells have many quality control systems to ensure that degraded/misfolded proteins do not accumulate within cells. Spontaneous errors in transcription and translation, and endogenous or exogenous oxidative stress, are among the most effective factors that may compromise proteome content. The more efficient the quality control systems are, the better the cells can survive noxious stimuli and preserve homeostasis. In particular, stem cells must have efficient quality control systems, as they reside for long periods within the niche of tissues of origin and may undergo several rounds of endogenous and exogenous injuries. Nevertheless, not all are created equal even among stem cells. Muse cells were discovered thanks to their powerful capacity to cope with stress, and we demonstrated that they tolerate extensive genotoxic stimuli better than MSCs and have an efficient DNA repair system [7,8].

The current findings suggest that in Muse cells, the quality control system that identifies and eliminates damaged proteins is highly active. Indeed, the great majority of GO terms enriched in Muse cells compared to the other analyzed cell phenotypes belong to vesicle trafficking, response to endoplasmic stress, and quality control of cellular components (Table 1, Table 2, Table 3). The content of the Muse cell proteome was also enriched in the endosome pathway, leading to MHC-I-mediated antigen processing presentation (Table 3). Proteolysis of endogenously synthesized proteins generates antigens that, loaded onto the cell surface together with MHC-I molecules, activate immune-surveillance by CD8 T cells. Any unfolded protein deriving from gene mutations or other stressful events can be processed and eliminated by an MHC-I-based system [30].

The capacity of Muse cells to cope with stress is also related to high reactive-oxygen-species (ROS)-scavenging activity. Indeed, many ontologies and pathways are related to oxidoreductase activity. Furthermore, some proteins exclusively identified in Muse cells have a potent antioxidant capacity, including SELONOM, SELENOBP, OXR1, and GPX8 [22,23,24,25]. 

The pathways related to cholesterol biosynthesis are enriched in the Muse cells proteome (Table 2). Cholesterol has a fundamental role in shaping cell membrane structure and in the function of membrane-localized proteins. In addition, the findings show that cholesterol is important for cell proliferation and cell survival; indeed, it contributes to radiation resistance and cell recovery after DNA damage with a still poorly known mechanism [31]. In this context, the enriched cholesterol biosynthesis pathways in Muse cells are in line with our previous finding showing Muse cells’ resistance to X-ray-induced DNA damage [7].

In a protein–protein interaction (PPI) network, a few, yet significant, nodes participate in up to hundreds of interactions (high-connectivity nodes) and hence have a fundamental role in many biological activities. Other nodes, with either a few or many interactions, connect the several subnetworks present in a complex network, such as PPI. These nodes have a high betweenness centrality and represent the obligate paths for connecting the functions associated with the different subnetworks. In this context, nodes with hundreds of interactions and a high betweenness centrality are of paramount importance for cell functions. We generated a Muse cell core network, having only nodes with the above-described features. This network further underlined the importance of the quality control program for Muse cells. In this network, we identified the proteins NFKB and CRKL. The first one is an almost ubiquitous transcription factor related to many biological processes. It can also have a role in protecting cells from apoptosis during exposure to a variety of stressors. CRKL is an adaptor protein and constitutes an integral part of the stress-activated protein kinase (SAPK) pathway.

In the other cell types, the enriched ontologies and pathways greatly differ from those identified in Muse cells, further emphasizing that active quality control is a peculiar characteristic of Muse cells. The proteome of iPSCs is enriched in ontologies and pathways that are related to cell division, DNA duplication, RNA transcription, and chromatin remodeling (Table 1, Table 2, Table 3). These data suggest that iPSCs are actively proliferating cells that need a full operativity of molecular mechanisms devoted to this task. It should be underlined that an active proliferation and duplication may reduce the quality control of biological processes and fidelity in the duplication of genetic material. In this context, the protein interaction analysis we performed on cells before and after stress treatment with peroxide hydrogen is of great interest. After DNA damage, cells exit the cycle to allow DNA repair. Then, cells can reenter a cell cycle if DNA has been repaired or, alternatively, cells undergo apoptosis or senescence if DNA is un- or misrepaired. After treatment with H_2_O_2_, Muse cells showed silencing of pathways that degrade CKIs earlier than iPSCs, suggesting that changes in the regulators of cell progression occur more promptly in Muse cells than in iPSCs (Figure 6A,B). Another interesting feature that differentiates Muse cells from iPSCs is the presence in the latter cells of pathways associated with the duplication of misrepaired DNA. Indeed, in iPSCs, there is activation of the TLS mechanism, which promotes DNA replication of damaged DNA strands through nucleotide misincorporation. The TLS avoids cell death but induces the accumulation of mutations. It can be a survival strategy of iPSCs in a hostile environment, but it can promote carcinogenesis. Indeed, iPSCs can produce teratomas both in vitro and in vivo [32]. At the opposite extreme, Muse cells that are nontumorigenic do not rely on TLS to survive [33].

## 4. Materials and Methods

### 4.1. Cultivation of MSCs

Adipose tissue-derived MSCs were obtained from the American Type Culture Collection (ATCC PCS-500-011) and were grown in DMEM containing 10% FBS (Fetal Bovine Serum), 4 mM L-glutamine, 100 U/mL penicillin-streptomycin, and 5 ng/mL bFGF. Cells were expanded until the 5th in vitro passage for proteome analysis and production of Muse and non-Muse cells (see below).

Flow cytometric characterization was performed by labeling cells with the following primary antibodies: CD34-PE, CD45-FITC, CD44-PE, CD73-APC, CD90-FITC, and CD105-PerCpCy5. The antibodies were used according to the manufacturer’s procedures (Santa Cruz Biotechnology, Dallas, TX, USA). After 30 min of incubation with the antibodies at room temperature, cells were washed with PBS and resuspended in FACS (Fluorescence Activated Cell Sorting) buffer for data acquisition on BD FACS Aria III device, and BD FACS Diva 8.0.1 program (Becton, Dickinson, Franklin Lakes, NJ, USA).

### 4.2. Isolation and Cultivation of Muse Cells and Non-Muse Cells

We isolated Muse cells by selecting the SSEA-3-positive population from MSCs with the MACS (Magnetic Activated Cell Sorting) method (Miltenyi Biotec, Auburn, CA, USA). In detail, PE-conjugated rat anti-SSEA-3 (MC631 clone) (BD Pharmingen, NJ, USA) primary antibody and mouse anti-PE microbead (Miltenyi Biotec) were used for sorting. MSCs (5 × 10^7^) were dissolved in 2 mL MACS Buffer (2 mM EDTA, 0.5% BSA), and 160 µL anti-SSEA-3-PE was added to the cell suspension. Cells were incubated for 20 min at 4 °C, then the suspension was centrifuged at 300× *g* for 10 min. The obtained pellet was dissolved in 300 µL MACS buffer. Then, anti-PE microbeads were added and the sample was incubated at 4 °C for 20 min. After incubation, the sample was centrifuged and the dissolved pellet was loaded onto LS columns (Miltenyi Biotec) for the MACS procedure, according to the manufacturer’s instructions. The collected SSEA-3-positive cells were Muse cells, while the negative fraction was designated as non-Muse cells.

MUSE cells were cultivated in suspension on poly-2-hydroxyethyl methacrylate (pHEMA)-coated Petri dishes, while non-MUSE cells were grown in normal flasks. Both SSEA-3-positive and -negative fractions were cultured in DMEM low-glucose medium containing 10% FBS 4 mM L-glutamine, 100 U/mL penicillin-streptomycin, and 5 ng/mL bFGF.

The spontaneous differentiation capacity of Muse cells was evaluated according to the published protocols [12]. In brief, Muse clusters from cultures in suspension were collected and dissociated mechanically into single cells. Cells were plated into 24-well culture dishes that were coated with 0.1% gelatin and grown for two weeks in α-MEM containing 10% FBS 4 mM L-glutamine and 100 U/mL penicillin-streptomycin. At the end of differentiation, cells were collected and RNA was extracted for evaluation of differentiation by RT-PCR.

### 4.3. Production and Cultivation of iPSCs

We used human dermal fibroblast cell lines (ATCC PCS-201-012) for production of iPSCs. Fibroblasts were cultured in a DMEM high-glucose medium containing 10% FBS, 4 mM L-Glutamine, and 100 U/mL penicillin-streptomycin. Semiconfluent cultures were transfected with episomal reprograming plasmid (System Biosciences, Mountain View, CA, USA) by using the Neon Transfection kit and the Neon Transfection system (Thermo Fisher, Waltham, MA, USA). We followed the manufacturers’ instructions to obtain reprogrammed fibroblasts. The Episomal iPSC Reprogramming integration-free plasmid that has a gene cluster containing Oct4, Sox2, Klf4, L-myc, Lin28, shRNA-p53, and miR302/367 reprogramming factors, plus a GFP marker for monitoring transfection efficiency was used. After transfection, cells were seeded in a 0.1% gelatin-coated Petri dish and cultivated for seven days. Then, cells were trypsinized and separated according to the SSEA-4 expression. In detail, 2 × 10^6^ cells were dissolved in 80 µL MACS buffer that was supplemented with 20 µL anti-SSEA-4-Microbead (Miltenyi Biotec). The samples were incubated for 15 min at 4 °C. After incubation, cells were loaded on MACS LS columns following the same procedure described for Muse cells (see above). The SSEA-4-positive cells, representing bona fide iPSCs, were grown on a feeder layer composed of mouse embryonic fibroblasts (mEFBs) (Thermo Fisher). 

The mEFBs were cultivated in DMEM containing 10% FBS, 4 mM L-glutamine, and 100 U/mL penicillin-streptomycin. Cells (80% confluence) were incubated with 312.5 ng/mL mitomycin C for 2.5 h to stop cell growth. The cells were then washed with PBS and used as a feeder layer. We seeded iPSCs on semiconfluent mEFBs and added a DMEM high-glucose medium that was supplemented with 10% Knockout Serum Replacement (KSR), 0.1 mM β-mercaptoethanol, 4 mM L-glutamine, 100 U/mL penicillin-streptomycin, nonessential amino acids, 10 µM ROCK inhibitor, and 4 ng/mL bFGF. The culture medium was changed every second day until the colonies reached to well-distinguished size (12–14 days). Passaging was done by treating cells with 500 µM EDT and seeding SSEA-4-positive cells on a new feeder layer.

### 4.4. Alkaline Phosphatase (AP) Assay

Cells were incubated for 20 min with 3.7% paraformaldehyde (PFA). After incubation, cells were treated for 10 min with NTMT (100 mM NaCl, 100 mM Tris HCl pH: 9.5, 50 mM MgCl, 1% Tween-20). Then, cells were incubated in the dark with a staining solution made of NBT (nitro-blue tetrazolium chloride) and BCIP (5-bromo-4-chloro-3’-indolyphosphate p-toluidine salt) (Roche-Thermo Fisher). Feeder cells were used as a negative control of AP assay.

### 4.5. Immunocytochemistry

We grew iPSCs in 35 mm dishes and then fixed them in 4% formaldehyde solution for 15 min at room temperature. We used the following primary antibodies: OCT4 (Abcam, UK) 1:200 (*v*/*v*); SOX2 (Abcam) 1:500 (*v*/*v*). The secondary antibodies (FITC conjugated) were obtained from Abcam. The SSEA-4-FITC and TRA-1-60-PE were fluorochrome-conjugated antibodies (Miltenyi Biotec, Bergisch Gladbach, Germany) that were diluted 1:50 (*v*/*v*). All of the above-described antibodies were used according to the manufacturer’s instructions. Nuclear staining was performed by a DAPI mounting medium (Abcam), and micrographs were taken under a Nikon Eclipse Ti Fluorescence Microscope (Nikon, Japan).

### 4.6. DNA Genotoxic Injury 

The iPSCs, MSCs, Muse cells, and non-Muse cells were incubated with 300 μM H_2_O_2_ for 30 min in a complete medium. The medium was then replaced with a fresh one, and the cells were incubated for 12, 24, or 48 h before further analysis.

### 4.7. Apoptosis Detection

The apoptosis was evaluated by the Annexin V test (Merck Millipore, Burlington, MA, USA) based on the measurement of phosphatidylserine (PS) externalization. The test was performed according to the manufacturer’s instructions. In brief, cells from the experimental groups were removed from culture dishes by trypsinization and stained with Annexin V-FITC solution containing 7-AAD. After the incubation, analyses were done using the BD FACS Aria III instrument (Becton, Dickinson). Evaluations were carried out using the BD FACS Diva 8.0.1 program.

### 4.8. Senescence Detection

Cells were fixed using a solution of 2% formaldehyde and 0.2% glutaraldehyde for 5 min at room temperature. After that, the cells were incubated with a staining solution containing 1 mg/mL of X-Gal (GoldBio, St. Louis, MO, USA) at 37 °C overnight. The percentage of senescent cells was calculated by the number of blue, beta-galactosidase-positive cells, out of at least 500 cells in different microscope fields, as already reported [34].

### 4.9. RT-PCR

RNA was extracted from cell samples with Omnizol (EuroClone, Pero (MI), Italy). The mRNA levels were determined by RT-PCR and by 5X All-In-One RT MasterMix (ABM, Richmond, BC, Canada), and the real-time PCR assays were carried out with BrightGreen 2X qPCR MasterMix (ABM, Vancouver, BC, Canada) and run on a LineGene 9600 PCR detection system (Bioer Technology, Hangzhou, China). All of the reagents were used according to the manufacturer’s instructions. We designed primer pairs for RT-PCR reactions with Primer Express software (Applied Biosystems, Milan, Italy) and used the mRNA sequences as templates from the Nucleotide DataBank (National Center for Biotechnology Information, Bethesda, MD, USA) to design primer pairs. Primer sequences are available upon request. We used the 2-ΔΔCT method as a relative quantification strategy for quantitative real-time PCR data analysis.

### 4.10. Proteome Isolation 

Cells obtained from trypsinization of cell cultures were collected and lysed according to Kulak’s method for efficient sample processing for proteomics [35]. In brief, cells were lysed with lysis buffer (6 M GdmCl, 40 mM CAA, 10 mM TCEP, 25 mM Tris-HCl pH 8.5). Proteins were digested with Trypsin/Lys-C and peptides were fractionated according to the InStage Digestion method using SDB-RPS disks. Peptides were eluted from the tip in three different fractions, which were dried in a vacuum centrifuge and re-dissolved with a solution containing 5% ACN and 0.1% formic acid.

### 4.11. LC-MS/MS Analysis

Samples were analyzed by reverse-phase HPLC-ESI-MS/MS using an Eksigent ekspert™ nanoLC 400 system (Eksigen, Dublin, CA, USA) directly connected to an AB SCIEX 5600+ TripleTOF mass spectrometer. Briefly, peptide mixtures were separated using the nanoACQUITY UPLC column (1.8 µM HSS T3 75 µM × 250 mm) (Waters Corp., Milford, MA, USA); 4 µL of sample was injected into the column and washed using 4% mobile phase B (0.1% (*v*/*v*) formic acid in 98% ACN) and 96% mobile phase A (0.1% (*v*/*v*) formic acid in 2% ACN) for 5 min water. The gradient ran from 4% B to 45.6% B over 150 min, and increased to 85% B to wash the column; finally, the column was equilibrated with 4% B. Peptides separated by LC were ionized using 15 units of Gas1 and 25 units of Curtain Gas, using 2400 V ISVF (ion source voltage frequency) and 75 °C IHT (interface heater temperature) conditions. HPLC-MS mode. Advanced data-dependent acquisition (DDA) was used for MS/MS collection with a dynamic collision energy setting for the 35 most abundant parent ions.

The collected MS data were searched against the human UniProtKB database (UP000005640 23 May 2019) by using Protein Pilot 4.5 Beta (Sciex GmbH, Darmstadt, Germany) on our server. The parameters were used for database searches include trypsin as a protease and Cys-alkylation and variable biological modifications. All peptide matches were initially filtered based on the Protein-Pilot peptide confidence (≥95%). Proteins were identified with two or more unique peptides at a 1% global false discovery rate (FDR) used for further analysis.

For every experimental condition, we performed two biological replicates, and for each of them, we performed two technical replicates. We utilized only the proteins that were consistently present in biological and technical replicates of each experimental condition. Further information can be found in supplementary files.

### 4.12. Gene ontology Analysis

The protein datasets obtained with LC-MS/MS analysis were analyzed using the PANTHER (http://www.pantherdb.org (accessed on 18 February 2021)) software. In PANTHER, the protein classification was performed according to the ontology terms: Cellular component, protein class, molecular function, biological processes, and pathway. For the PANTHER analysis, we used the statistics overrepresentation (default setting), comparing classifications of multiple clusters of lists to a reference list in order to statistically identify the overrepresentation of PANTHER ontologies. The selected *p*-value was set at 0.05. We followed the developers’ instructions for running a PANTHER analysis [36].

### 4.13. Pathway Analysis

Differentially expressed proteins were imported into Reactome software for detailed pathway identification [13,14]. The Reactome Knowledgebase (https://reactome.org (accessed on 18 February 2021)) provides molecular details of cellular processes as an ordered network of molecular transformations in a single consistent data model. We submitted LC/MS data as a single column of identifiers (UniProt IDs), and the software mapped them to pathways. Overrepresentation and pathway-topology analyses were conducted. Overrepresentation analysis is based on statistical hypergeometric distribution, and it evaluates whether certain specific Reactome pathways are enriched in the submitted data. This analysis produced a probability score, which was then corrected for false discovery rate (FDR) using the Benjamini–Hochberg method. The FDR was set at *p* < 0.05.

### 4.14. Network Analysis

The protein–protein interaction network (PPI) was evaluated by importing protein datasets, which we obtained with LC-MS/MS analysis, into NetworkAnalyst online software (www.networkanalyst.ca (accessed on 18 February 2021)). NetworkAnalyst is a comprehensive gene expression analysis, meta-analysis, and network biology software. To create a network, we used the IMEX interactome with default FDR significance. Having large datasets, we reduced the network size by selecting the Minimum Network to build minimally connected networks. These networks contained all of the seed genes/proteins, and the only added nodes are those that connect previously disjointed networks of seed genes/proteins. For the minimum network, the software computes pair-wise shortest paths between all seed nodes and removes the nodes that are not on the shortest paths.

## 5. Conclusions 

Our current findings, together with our previous investigation of DNA repair capacity, have evidenced that Muse stem cells have an enduring stress capacity that can be seen as a quick and effective quality control system that checks and eliminates damaged macromolecules (proteins and nucleic acids). This property may promote Muse cells as an attractive natural alternative to iPSCs in cell therapy. Further studies have to dissect in-depth the molecular mechanisms that we evidenced with our proteome analysis of Muse cells. In particular, silencing experiments of key nodes identified in the core network of Muse cells, as well as the inactivation of some ROS scavengers identified in the Muse proteome, will add further knowledge of the complex system Muse cells adopt to preserve their biological properties.

## Figures and Tables

**Table 1:** Excerpt of cell-type-specific gene ontology (GO) ontologies.

iPSCs	MSCs	Muse Cells	Non-Muse Cells
**cell-type-specific GO biological process**			
chromatin organization	organelle membrane fusion	response to topologically incorrect protein	chemokine-mediated signaling pathway
chromosome organization	organelle fusion	regulation of endocytosis	cellular response to chemokine
histone modification	vesicle fusion	ubiquitin-dependent ERAD pathway	response to chemokine
DNA repair	COPII-coated vesicle budding	response to nitrogen compound	
DNA replication	proteasome assembly	response to endoplasmic reticulum stress	
DNA biosynthetic process		regulation of proteolysis	
DNA-dependent DNA replication		cellular response to topologically incorrect protein	
DNA unwinding involved in DNA replication			
DNA strand elongation involved in DNA replication			
RNA 3’-end processing			
mRNA 3’-end processing			
RNA modification			
tRNA metabolic process			
maturation of 5.8S rRNA			
mRNA polyadenylation			
mitochondrial RNA metabolic process			
rRNA modification			

**Table 2:** Excerpt of cell-type-specific GO ontologies.

iPSCs	MSCs	Muse Cells	Non-Muse Cells
**cell-type-specific GO molecular function**			
RNA polymerase activity	ubiquitin-like protein ligase binding	ubiquitin protein ligase activity	ammonium ion binding
catalytic activity, acting on DNA	ubiquitin protein ligase binding	oxidoreductase activity	nucleoside-triphosphatase regulator activity
RNA methyltransferase activity		sterol binding	sodium ion transmembrane transporter activity
RNA polymerase III activity		protease binding	
exonuclease activity		peptidase regulator activity	
nuclease activity			
histone binding			
single-stranded DNA binding			
DNA-dependent ATPase activity			
3’-5’ DNA helicase activity			
5’-3’ RNA polymerase activity			
DNA-directed 5’-3’ RNA polymerase activity			
**cell-type-specific GO pathways**			
DNA replication	Serine glycine biosynthesis	Inflammation mediated by chemokine and cytokine signaling pathway	VEGF signaling pathway
De novo pyrimidine ribonucleotides biosythesis		Insulin/IGF pathway-mitogen-activated protein kinase kinase/MAP kinase cascade	
Succinate to proprionate conversion		Pyruvate metabolism	
		ATP synthesis	
		Cholesterol biosynthesis	
		Pyrimidine Metabolism	

**Table 3:** Cell-type-specific pathways identified by Reactome analysis.

iPSCs	MSCs	Muse Cells	Non-Muse Cells
Lagging Strand Synthesis	SCF(Skp2)-mediated degradation of p27/p21	Endosomal/Vacuolar pathway	RNA Polymerase II Transcription Initiation
Leading Strand Synthesis	Mitochondrial calcium ion transport	ER-Phagosome pathway	
Polymerase switching on the C-strand of the telomere	RAB geranylgeranylation	Antigen processing-Cross presentation	
Polymerase switching		Antigen Presentation: Folding, assembly, and peptide loading of class I MHC	
Resolution of Sister Chromatid Cohesion		Class I MHC-mediated antigen processing and presentation	
Mitotic Anaphase		Post-chaperonin tubulin folding pathway	
Gap-filling DNA repair synthesis and ligation in GG-NER		Platelet degranulation	
Dual incision in TC-NER		DNA Damage Recognition in GG-NER	
Recognition of DNA damage by PCNA-containing replication complex		RHO GTPases activate KTN1	
Small interfering RNA (siRNA) biogenesis			
Glucose metabolism			
Transport of connexons to the plasma membrane			
Microtubule-dependent trafficking of connexons from Golgi to the plasma membrane			
RHO GTPases activate IQGAPs			

**Table 4:** Key nodes with degree > 100 and betweenness > 10,000.

iPSCs	MSCs	Muse Cells	Non-Muse Cells
UBC	CDK2	UBC	UBC
CUL3	CUL3	CUL3	CUL3
SUMO2	SUMO2	ELAVL1	SUMO2
APP	KIAA0101	FN1	KIAA0101
KIAA0101	FN1	KIAA0101	HNF4A
HNF4A	HNF4A	SUMO2	ELAVL1
FN1	APP	COPS5	APP
ELAVL1	ELAVL1	HNF4A	FN1
SIRT7	COPS5	APP	COPS5
CAND1	CAND1	CAND1	CAND1
COPS5	ESR1	ESR1	SIRT7
ESR1	SIRT7	YWHAZ	ESR1
YWHAZ	YWHAZ	SIRT7	YWHAZ
CDK2	CUL1	ITGA4	CDK2
ITGA4	CDK2	VCAM1	ITGA4
CUL1	ITGA4	CDK2	CUL1
MYC	GRB2	CUL1	GRB2
SUMO1	SUMO1	SUMO1	MYC
IKBKE	MYC	MYC	SUMO1
TRAF6	IKBKE	IKBKE	IKBKE
UBD	UBD	CUL2	VCP
VCP	TRAF6	VCP	UBD
GRB2	CUL2	GRB2	CUL2
HSP90AB1	VCP	UBD	TRAF6
HDAC1	HSP90AB1	HSP90AB1	CUL5
EP300	YWHAQ	EGFR	HSP90AB1
SIRT1	HDAC1	YWHAQ	VHL
	EEF1A1	EEF1A1	YWHAQ
	IKBKG	HSPA8	UBL4
	HSPA8	PSMA3	HDAC1
	HDAC2	YWHAG	IKBKG
	YWHAG	AKT1	EEF1A1
	SMAD3	NFKB1	TP53
	PIK3R1		HSPA8
	CRK		YWHAG
	SHC1		SIRT1
	NCK1		AKT1
			HDAC2
			SMAD3
			HSPA4
			PXN
			CRK
			HNRNPA1
			JUN
			PIK3R1
			NCK1
			UBE2I
			VIM
			STAT1
			POLR2A
			PPP1CA

## Data Availability

Data are reported in the text and in supplementary files.

## Supplementary Materials

The following are available online at https://www.mdpi.com/1422-0067/22/4/2064/s1. Supplementary File 1: Flow cytometry analysis of MSC cultures. Flow cytometric characterization was performed by labeling MSCs with the following primary antibodies: CD34-PE, CD45-FITC, CD44-PE, CD73-APC, CD90-FITC, and CD105-PerCpCy5. The antibodies were used according to the manufacturer’s procedures (Santa Cruz Biotechnology, Dallas, TX, USA). After 30 min of incubation with the antibodies at room temperature, cells were washed with PBS and resuspended in FACS buffer for data acquisition on a BD FACS Aria III device and BD FACS Diva 8.0.1 program. The histograms show the flow cytometry reports. Supplementary File 2: Characterization of iPSCs. Panels (**A**–**D**): The first column shows representative FITC immunostaining for OCT3/4, SOX2, SSEA-4 (panels **A**–**D**) and PE immunostaining for TRA-1-60 (panel **C**). The second column shows the corresponding nuclear DAPI staining, and the last columns the merged images. Supplementary File 3: List of proteins identified in cell lysates. The spreadsheet named Proteome content shows the list of proteins found in iPSCs, MSCs, Muse cells, and non-Muse cells, either in basal conditions or following H_2_O_2_. For each cell type, there were two biological replicates (A, B), and each column shows proteins present in both the replicates. Proteins were listed with their UniProt identifiers. The spreadsheet named “Venn analysis of proteome content” shows the result of Venn analysis performed on proteins from iPSCs, MSCs, Muse cells, and non-Muse cells. Supplementary File 4: GO analysis carried out with PANTHER. The spreadsheets show ontology terms overrepresented in iPSCs, MSCs, Muse cells, and non-Muse cells. Ontology terms were classified as cellular components, protein classes, molecular functions, biological processes, and pathways. Supplementary File 5: Venn analysis on GO ontologies. The spreadsheets show both cell-type-specific and common ontology terms overrepresented in iPSCs, MSCs, Muse cells, and non-Muse cells. Ontology terms were classified as cellular components, protein classes, molecular functions, biological processes, and pathways. Supplementary File 6: Reactome analysis. The spreadsheets show the pathways identified in iPSCs, MSCs, Muse cells, and non-Muse cells by Reactome analysis, which was carried out on proteomes from control conditions at 12, 24, and 48 h following H_2_O_2_ treatment. Supplementary File 7: Protein–protein interaction minimum networks. The spreadsheets show the nodes present in minimum IMEx networks generated with NetworkAnalyst software by inserting the proteome data from iPSCs, MSCs, Muse cells, and non-Muse cells, Supplementary File 8: Reactome analysis of overrepresented pathways. Reactome analysis of protein datasets obtained from iPSCs, MSCs, Muse cells, and non-Muse cells before and after H_2_O_2_ treatment. Data are shown by Voronoi tessellation, which gives a general pathways overview. The overrepresented pathways (*p* < 0.05) are depicted in yellow.
