# Supplementary figures and images for: Why Do Muse Stem Cells Present an Enduring Stress Capacity? Hints from a Comparative Proteome Analysis
Source: Int J Mol Sci. 2021 Feb 19;22(4):2064. doi: 10.3390/ijms22042064 (PMC7922977; doi:10.3390/ijms22042064)

# BD FACSDiva 8.0.1

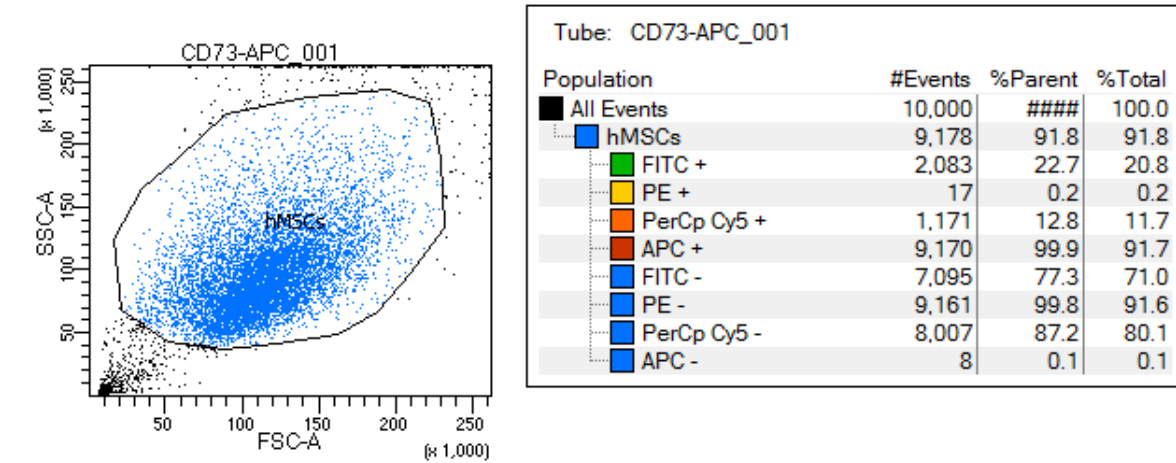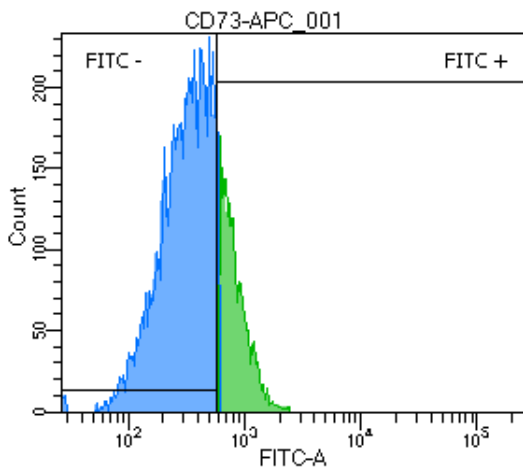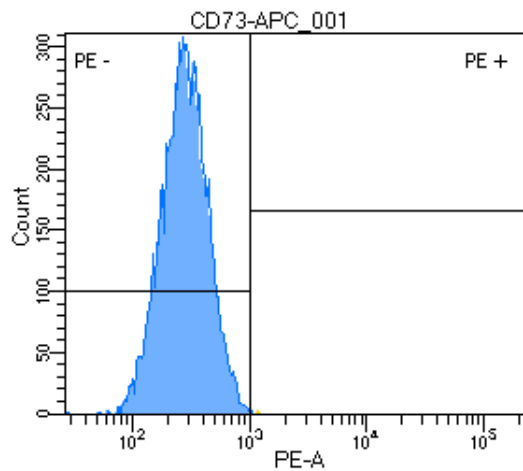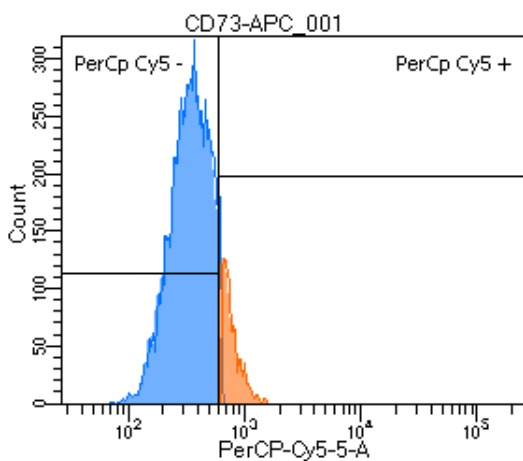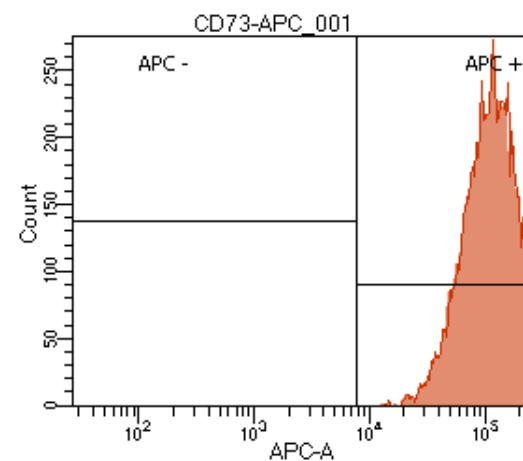

# BD FACSDiva 8.0.1

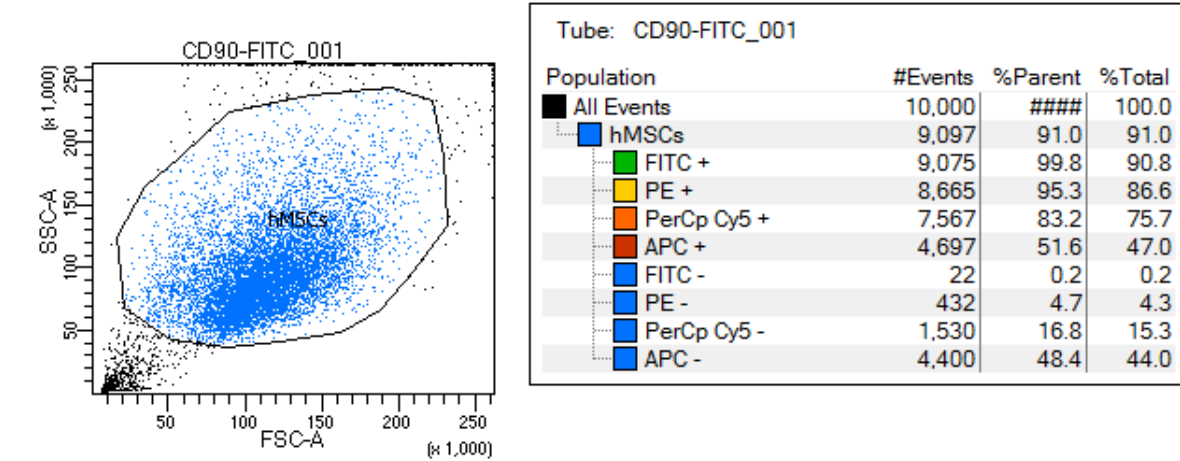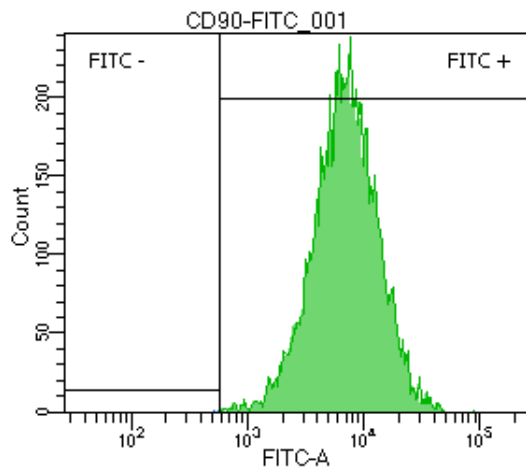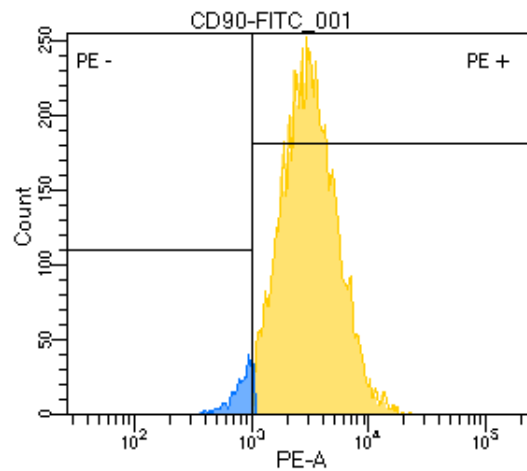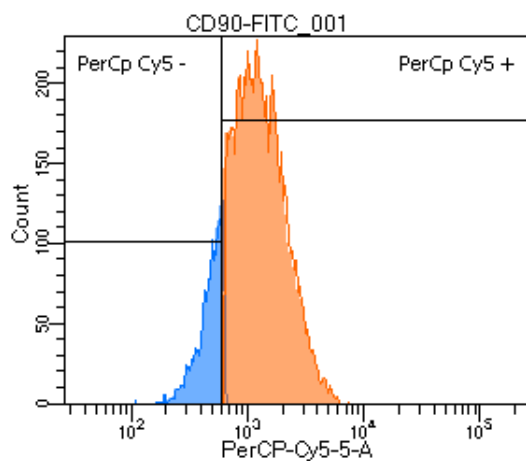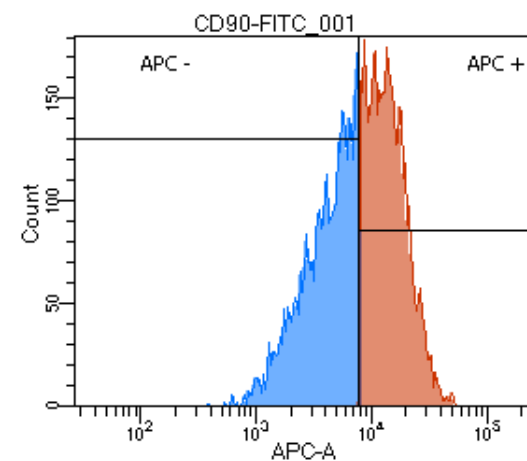

# BD FACSDiva 8.0.1

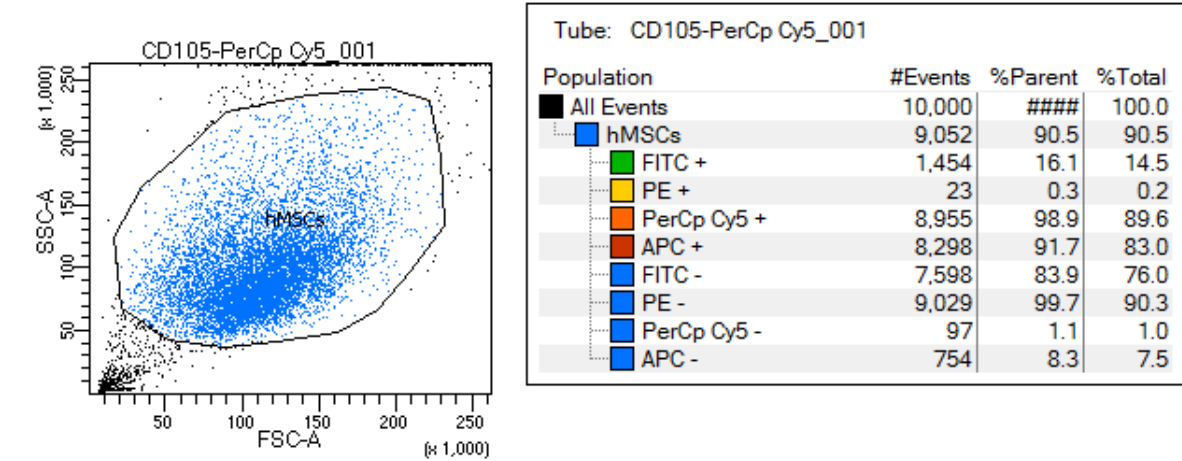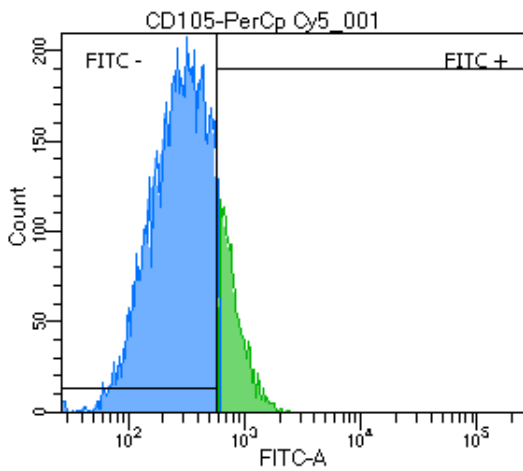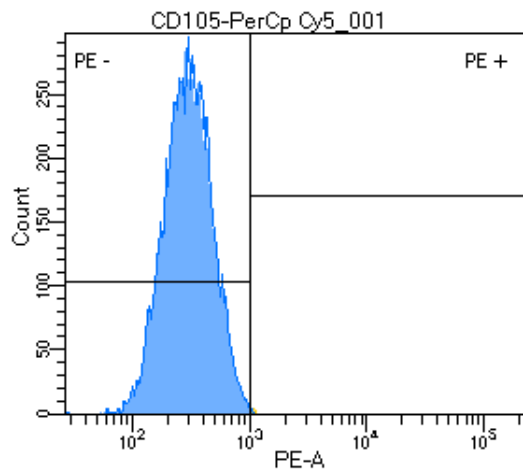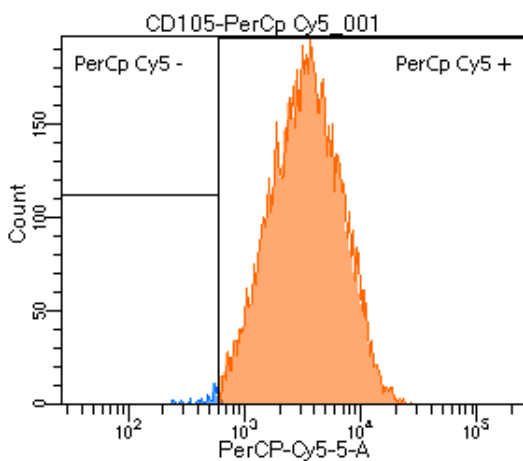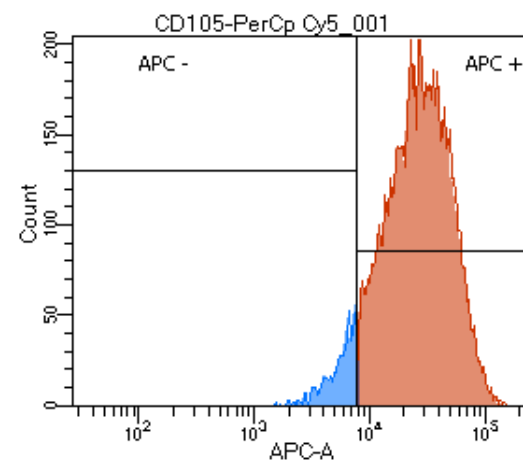

# BD FACSDiva 8.0.1

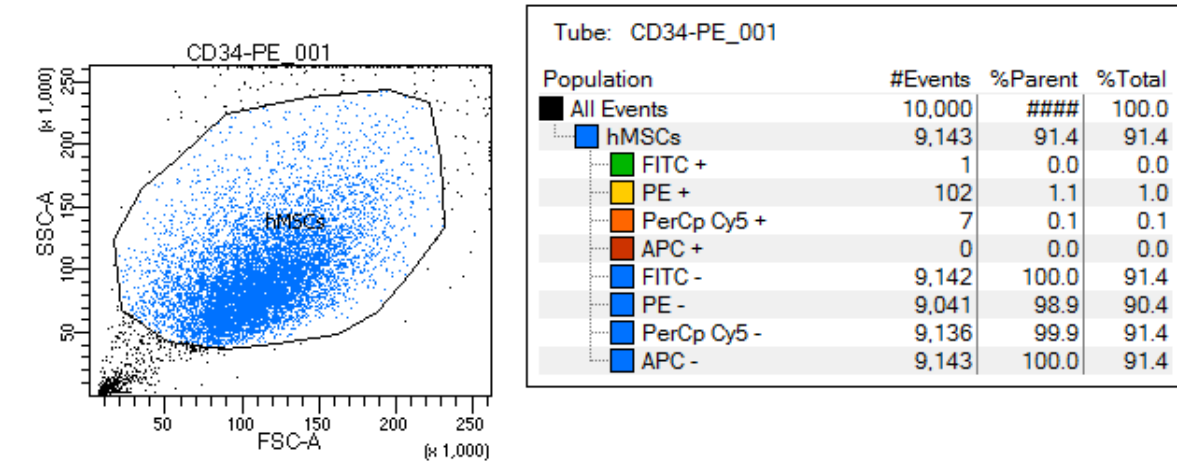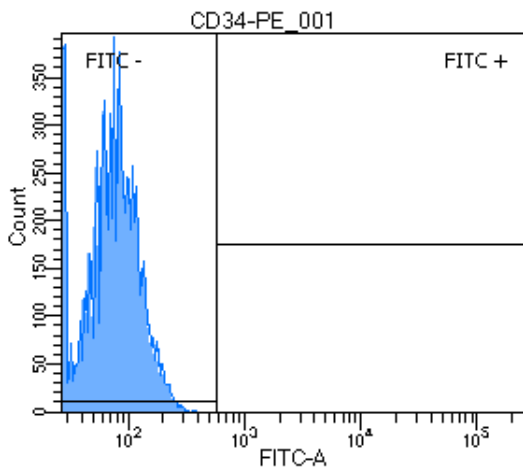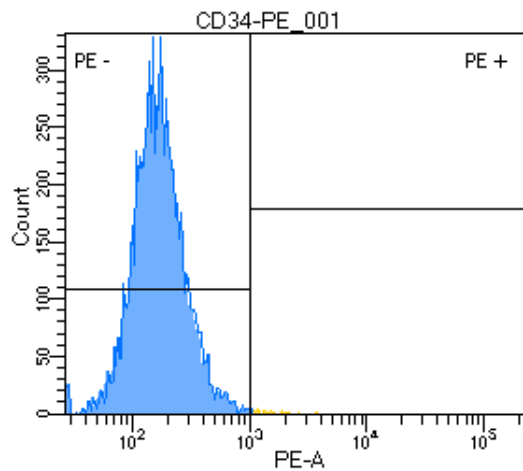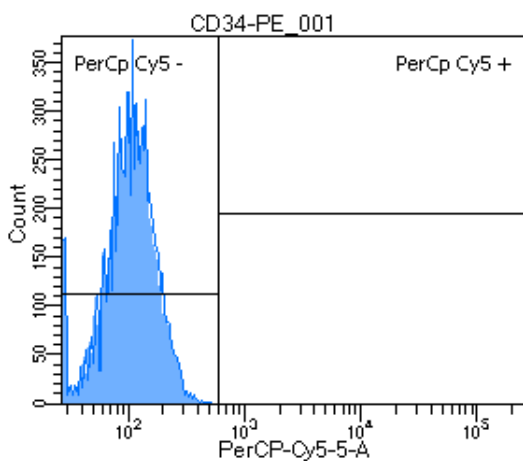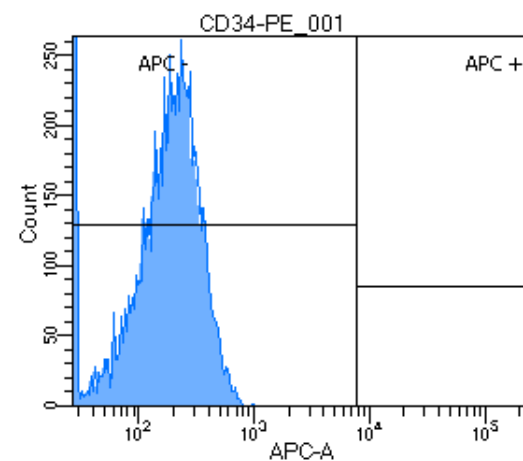

# BD FACSDiva 8.0.1

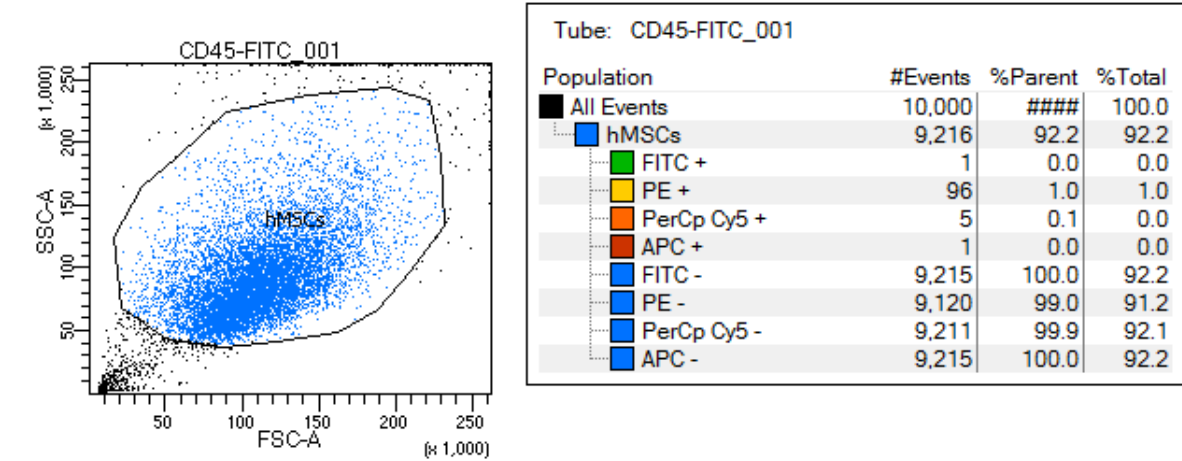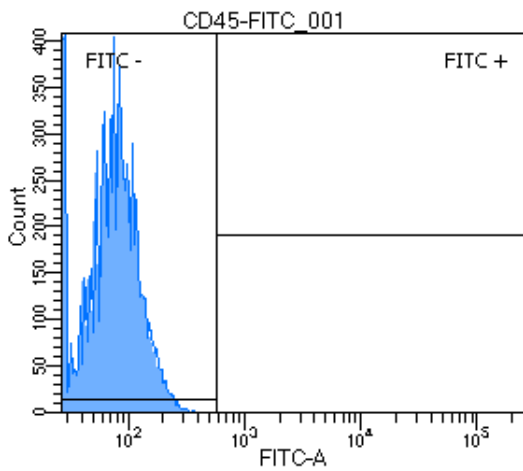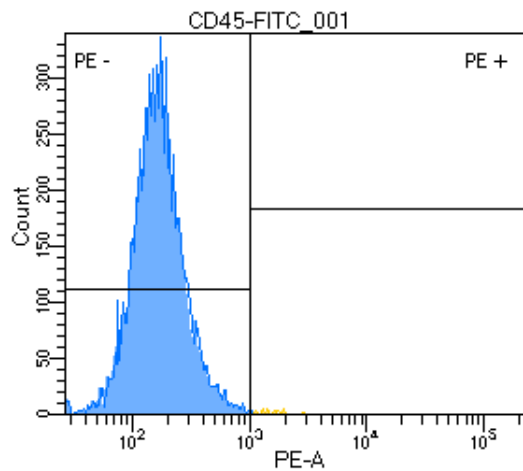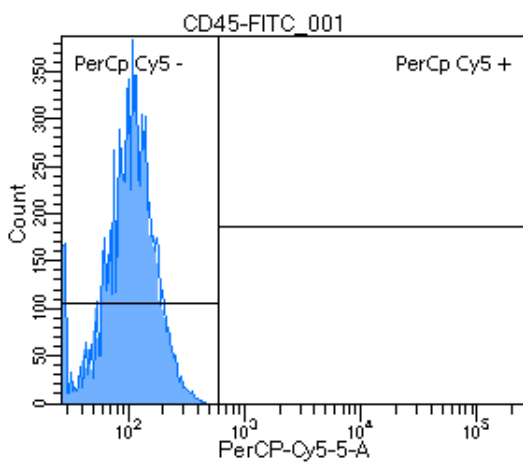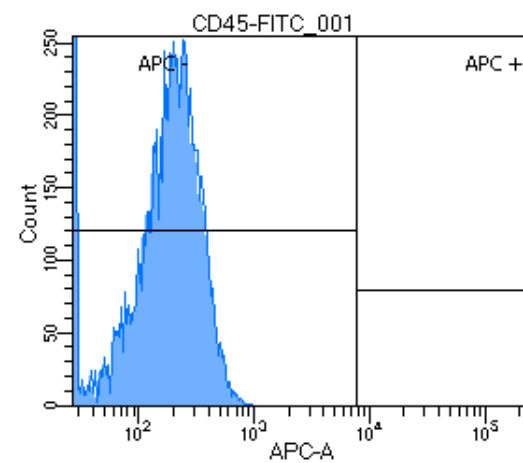

Supplement: Supplementary file 1 [file ijms-22-02064-s001.zip › Supplementary file 1.pdf]

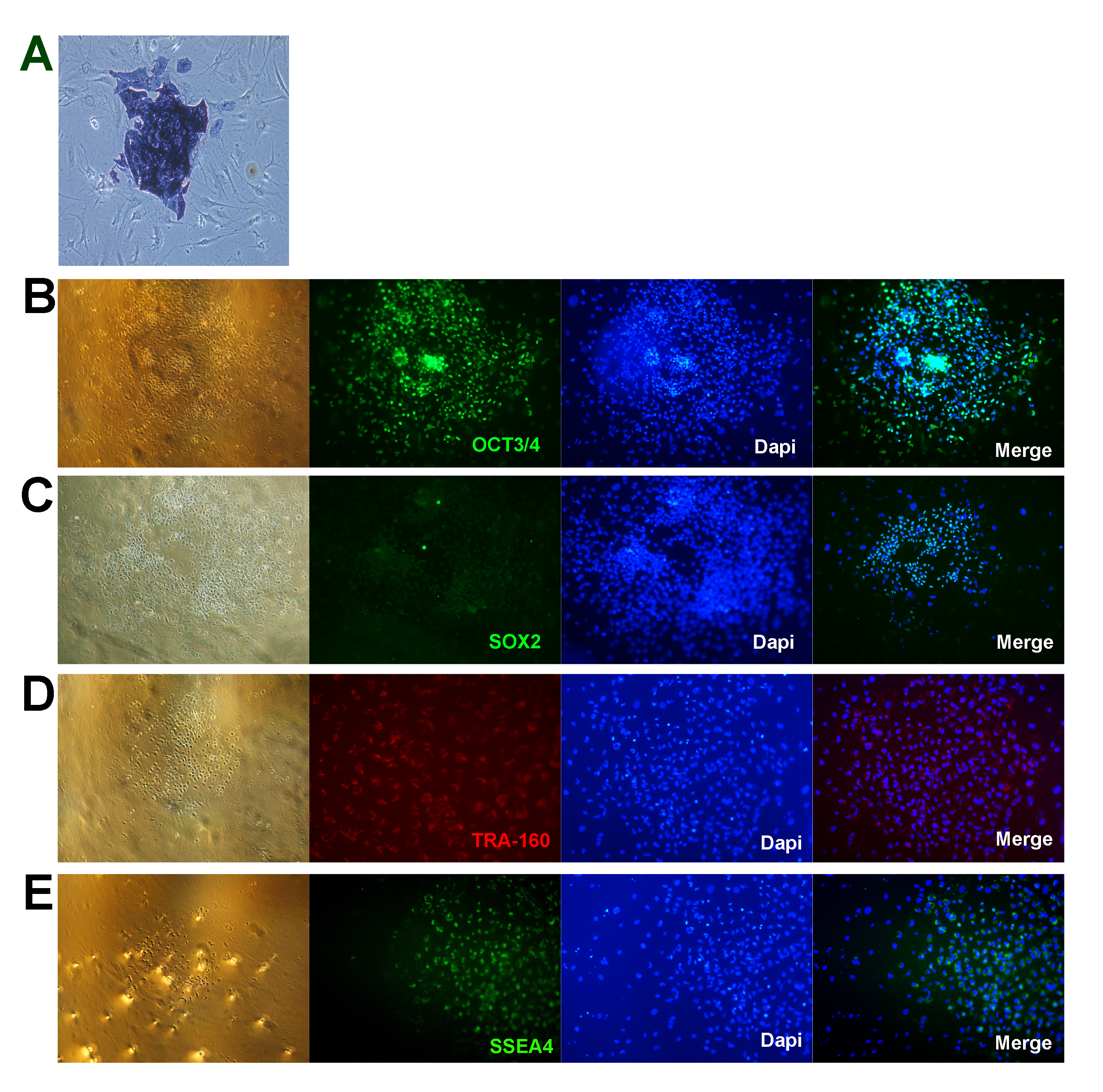

Supplement: Supplementary file 1 [file ijms-22-02064-s001.zip › Supplementary file 2.tif]

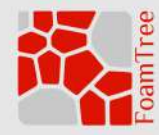

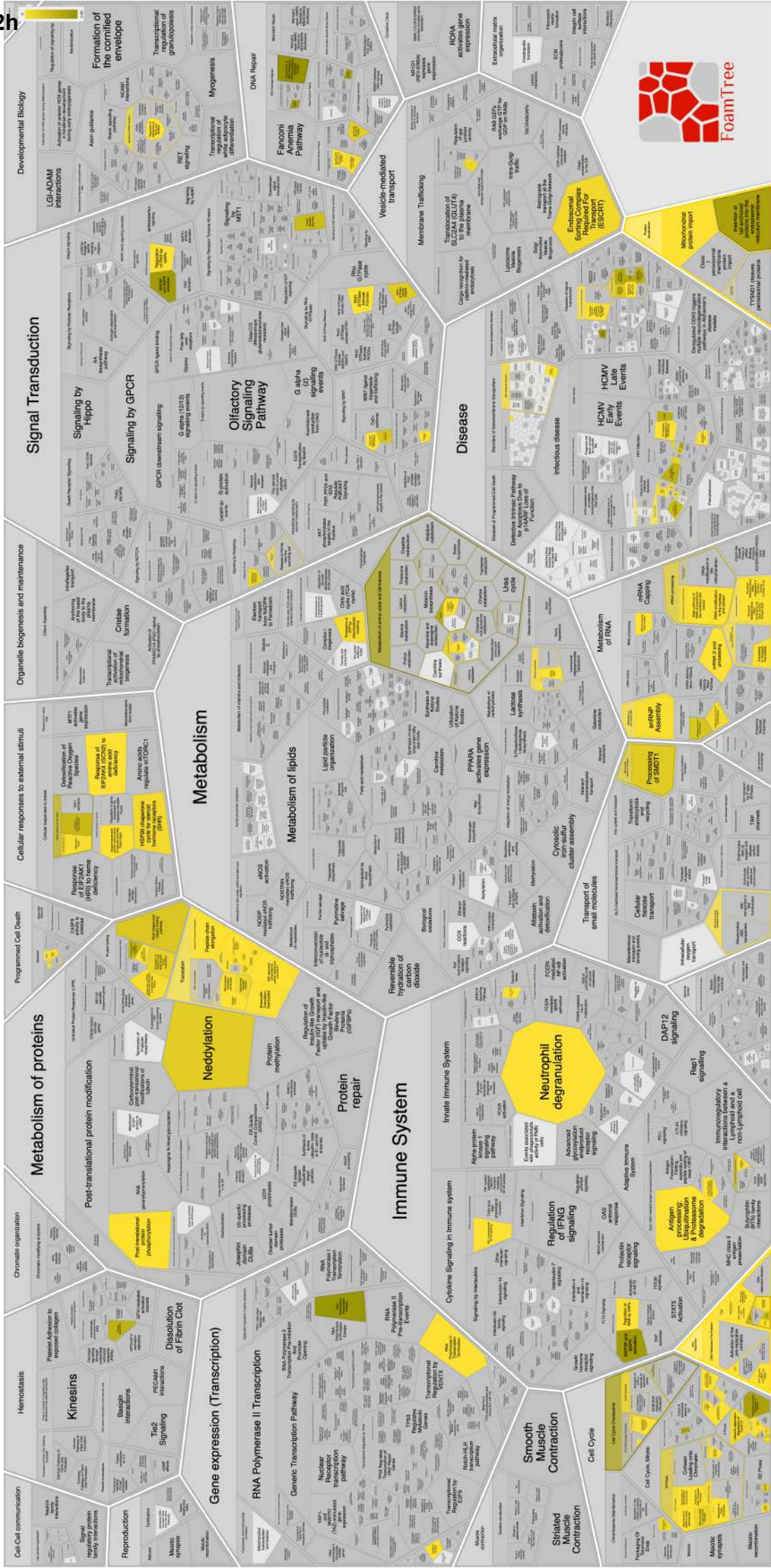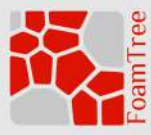

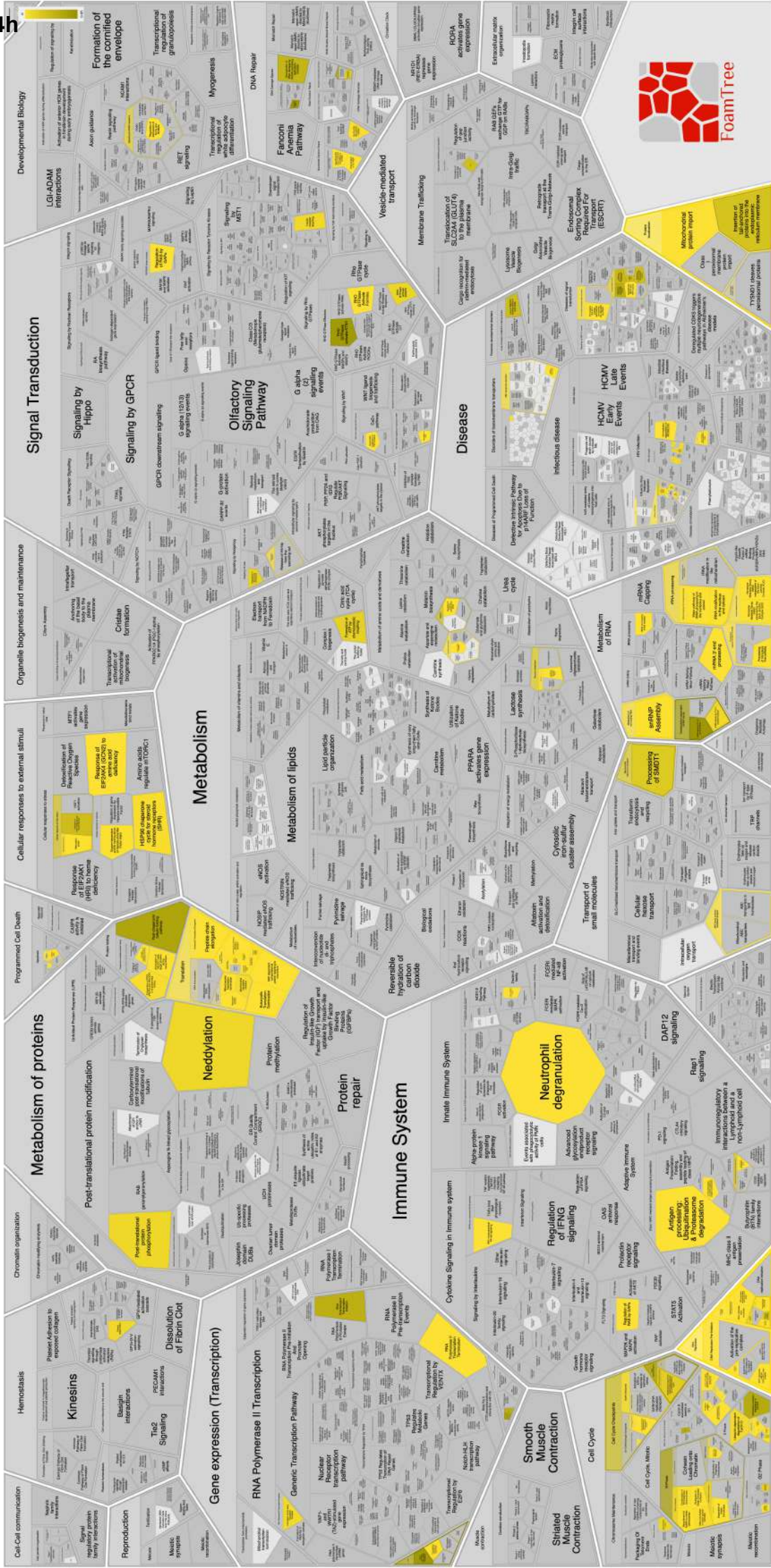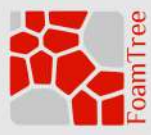

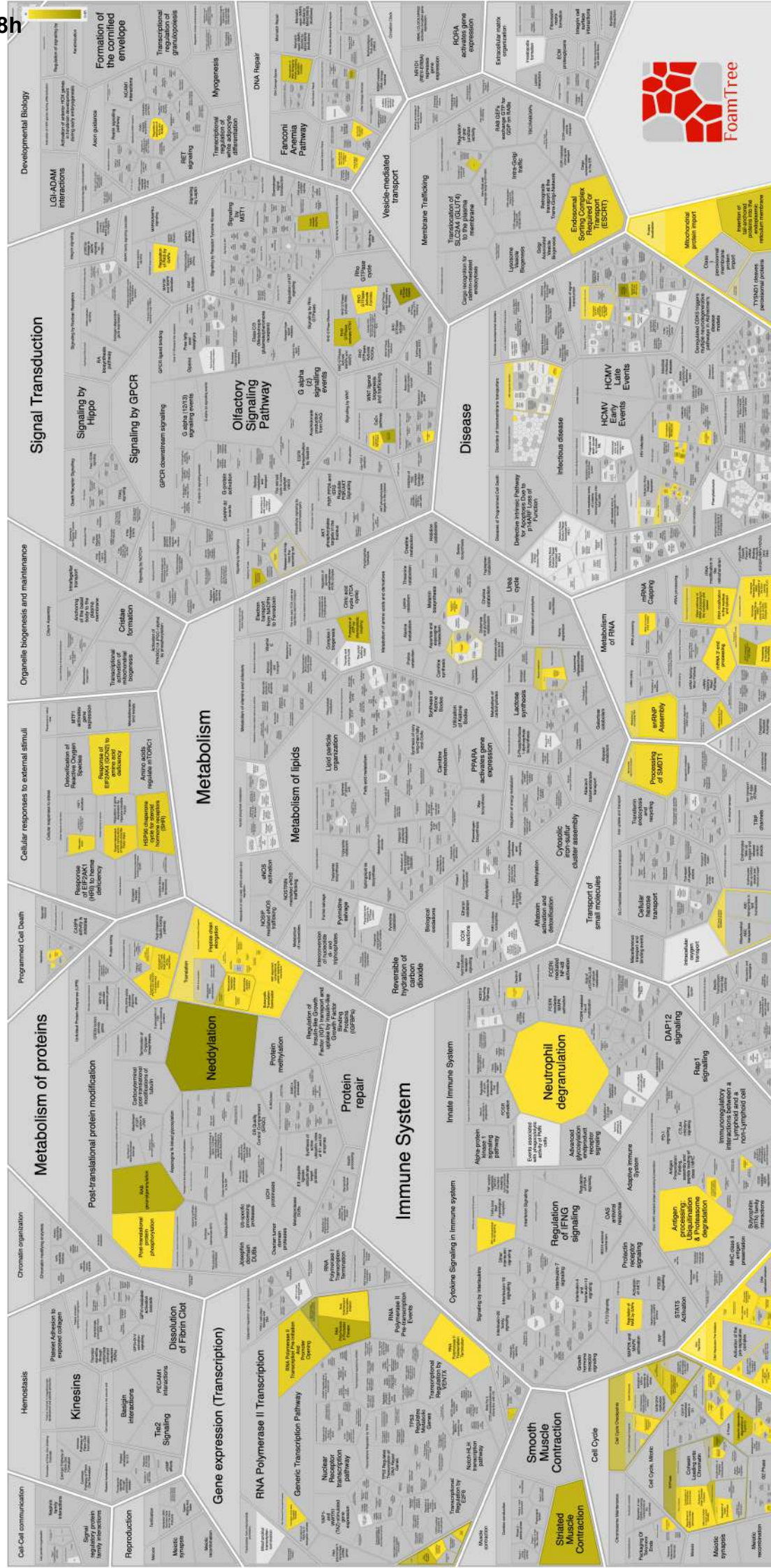

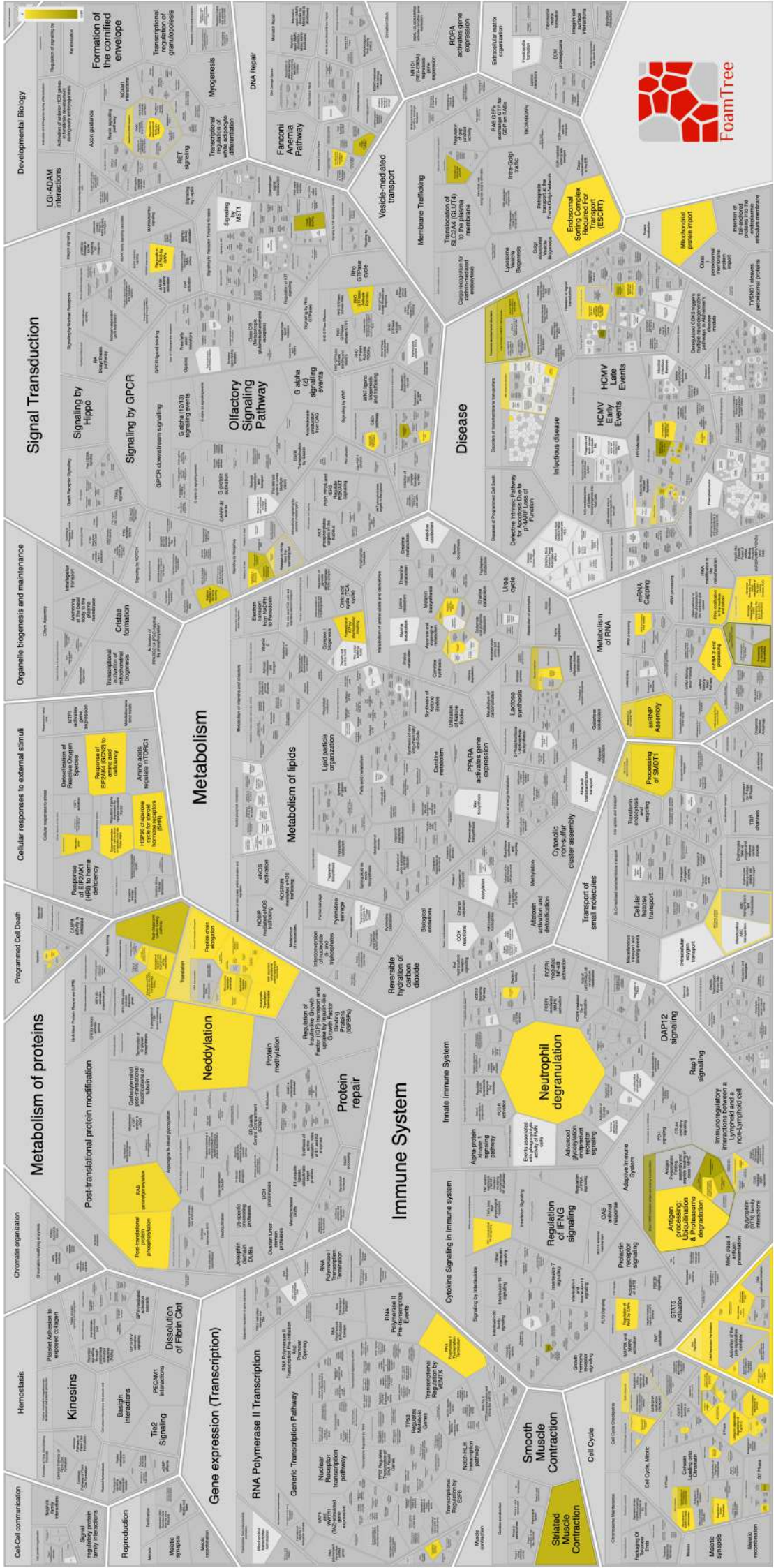

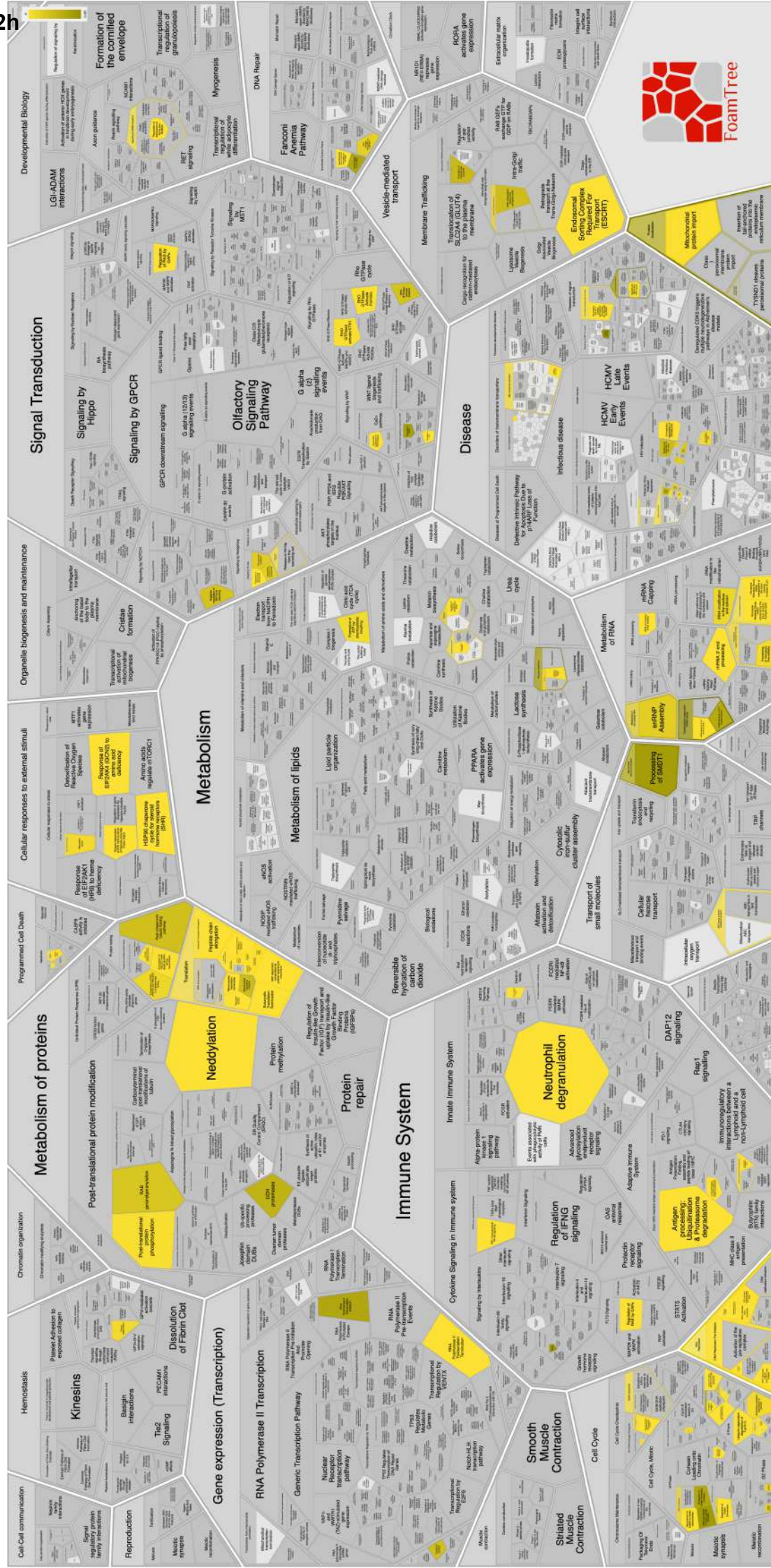

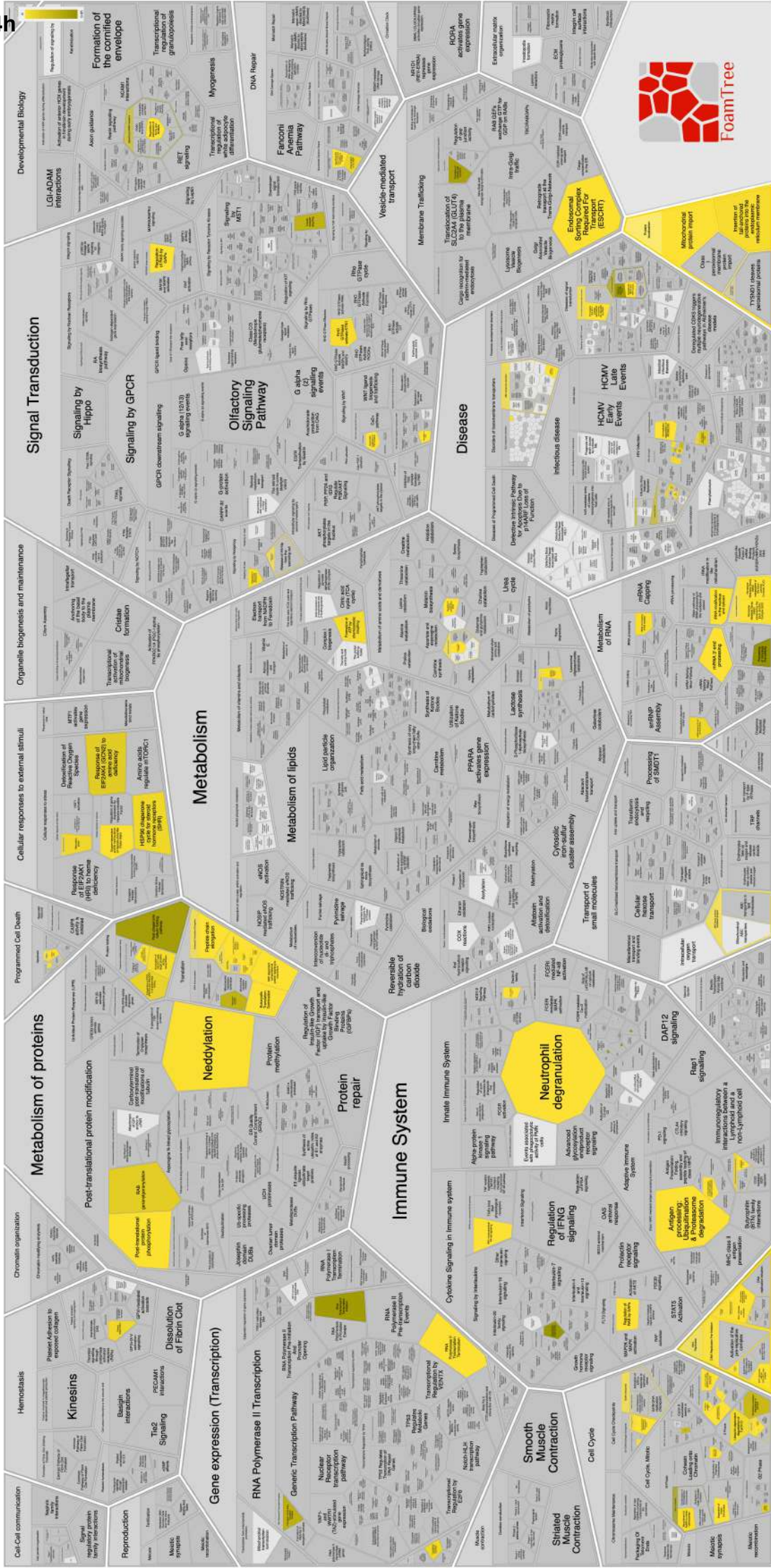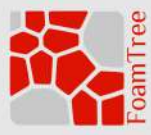

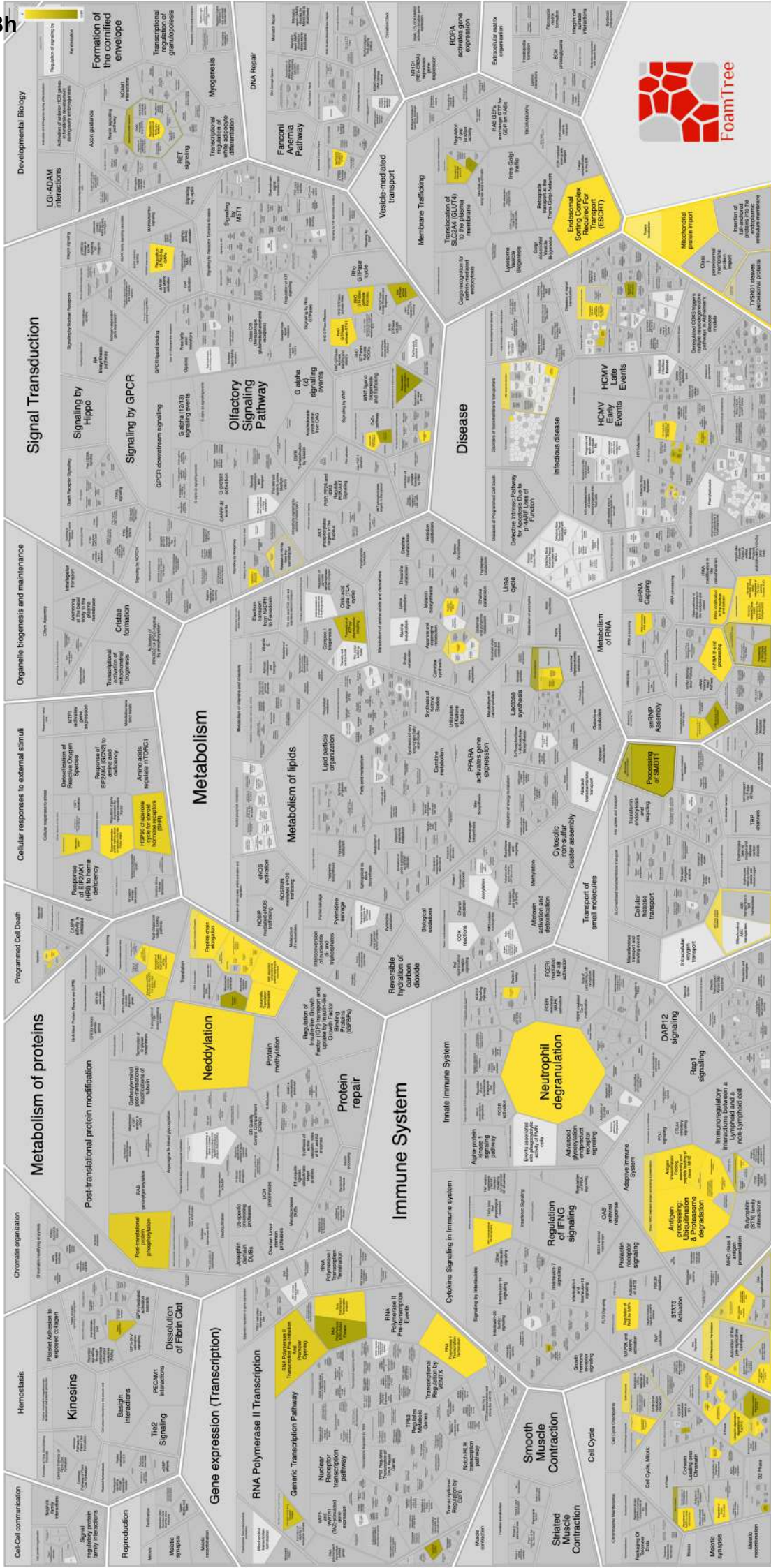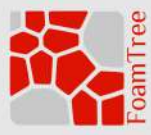

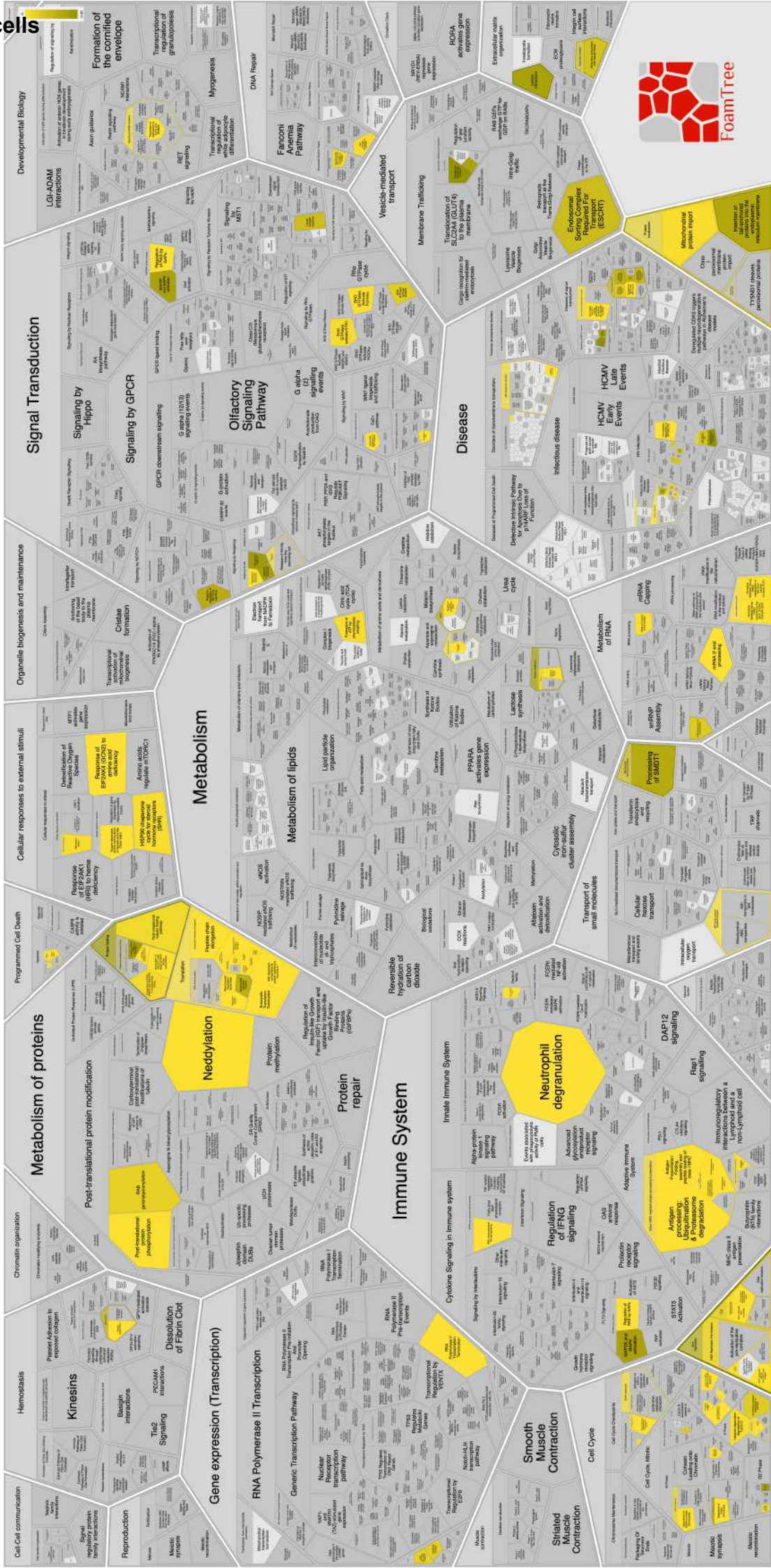

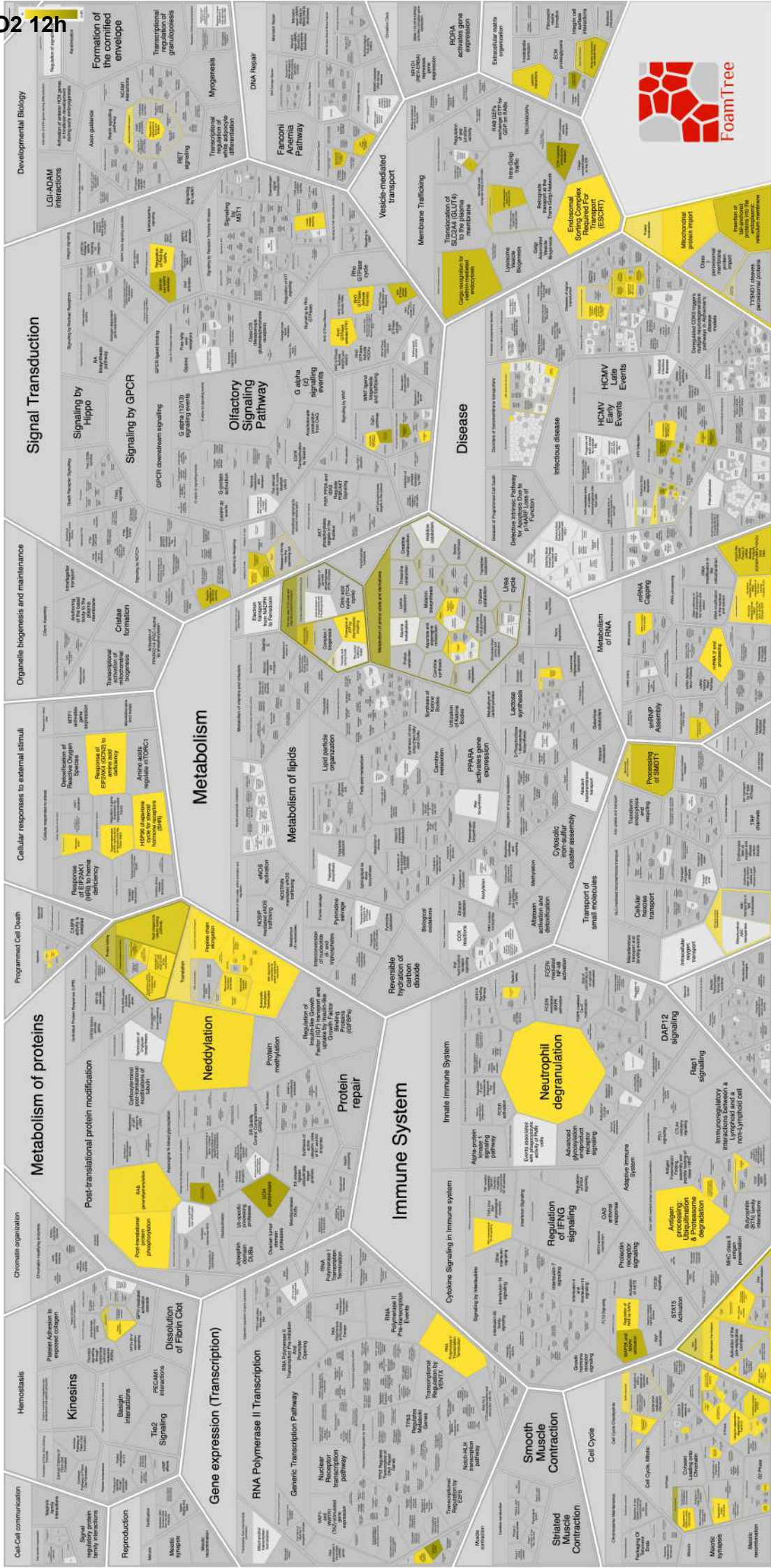

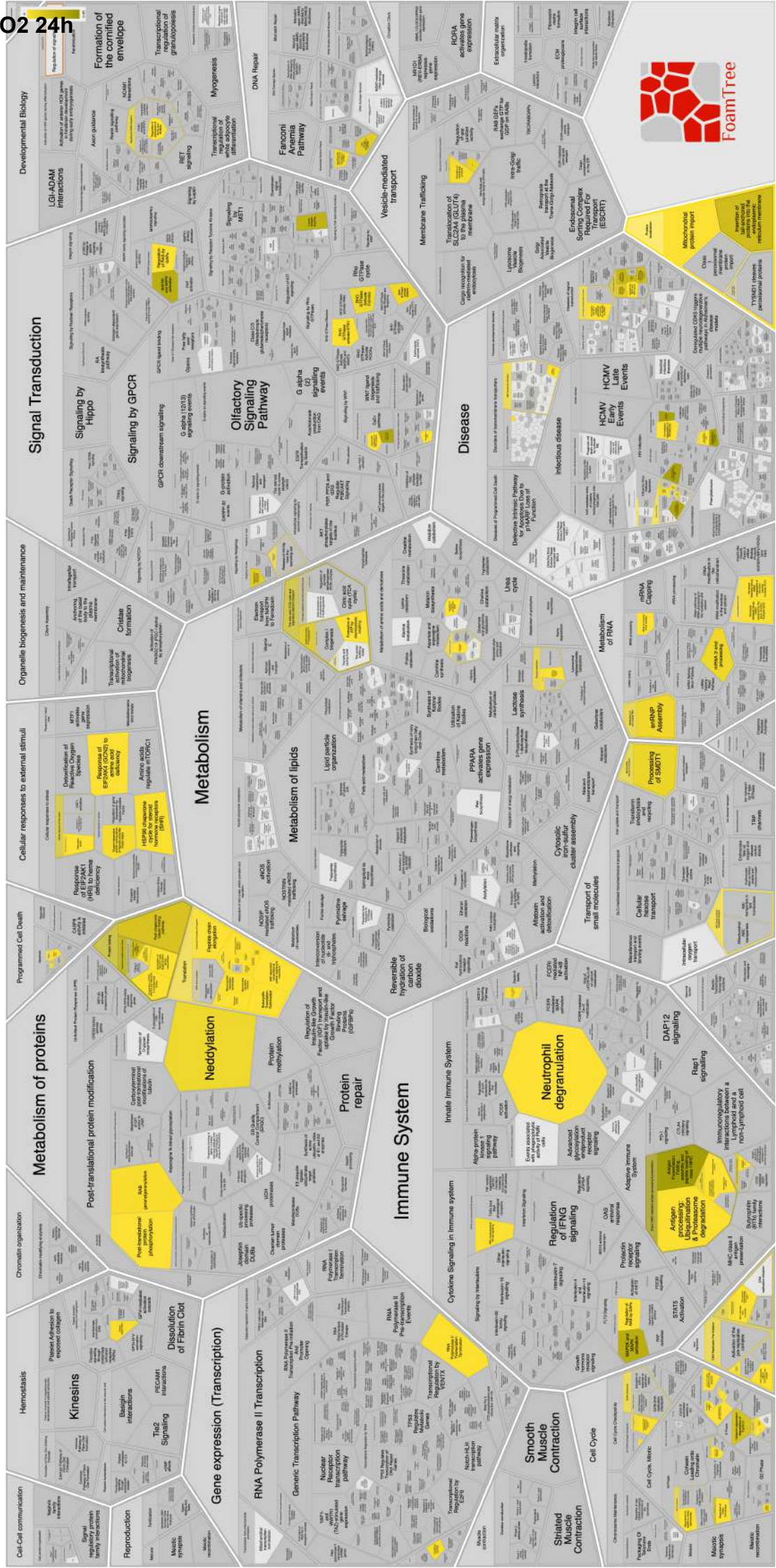

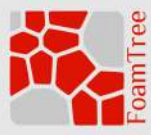

control Non-Muse cells

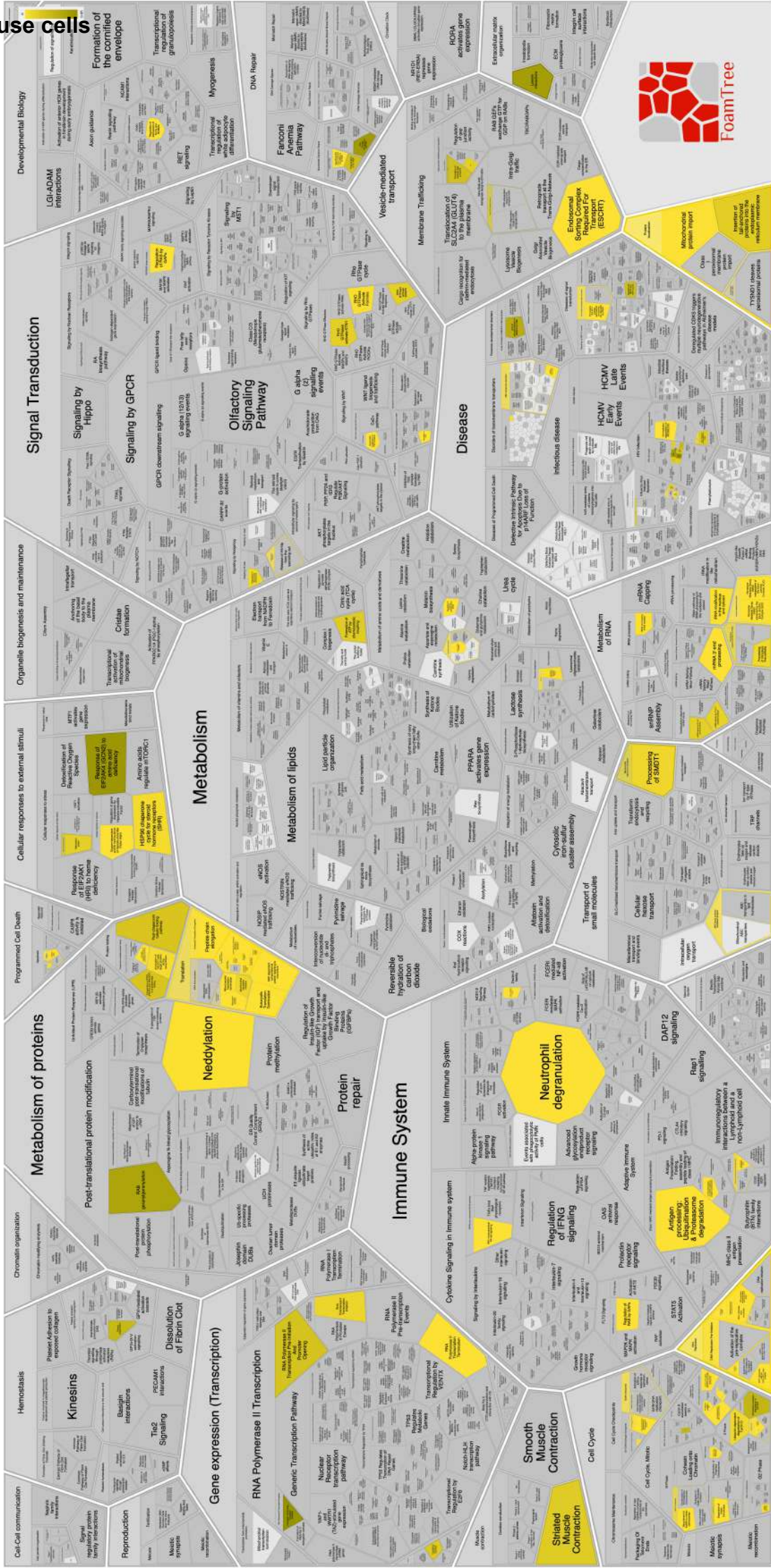

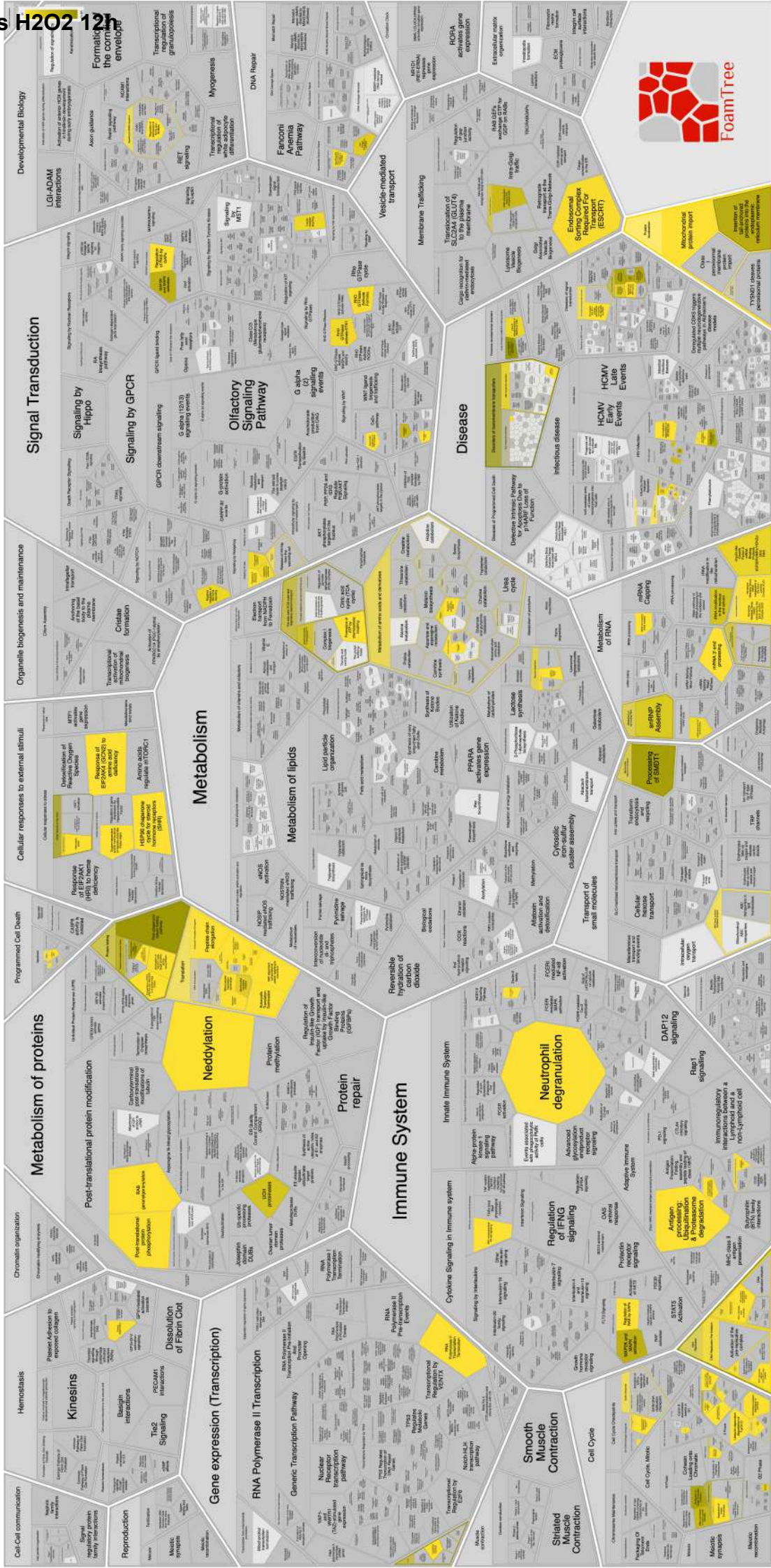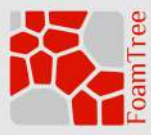

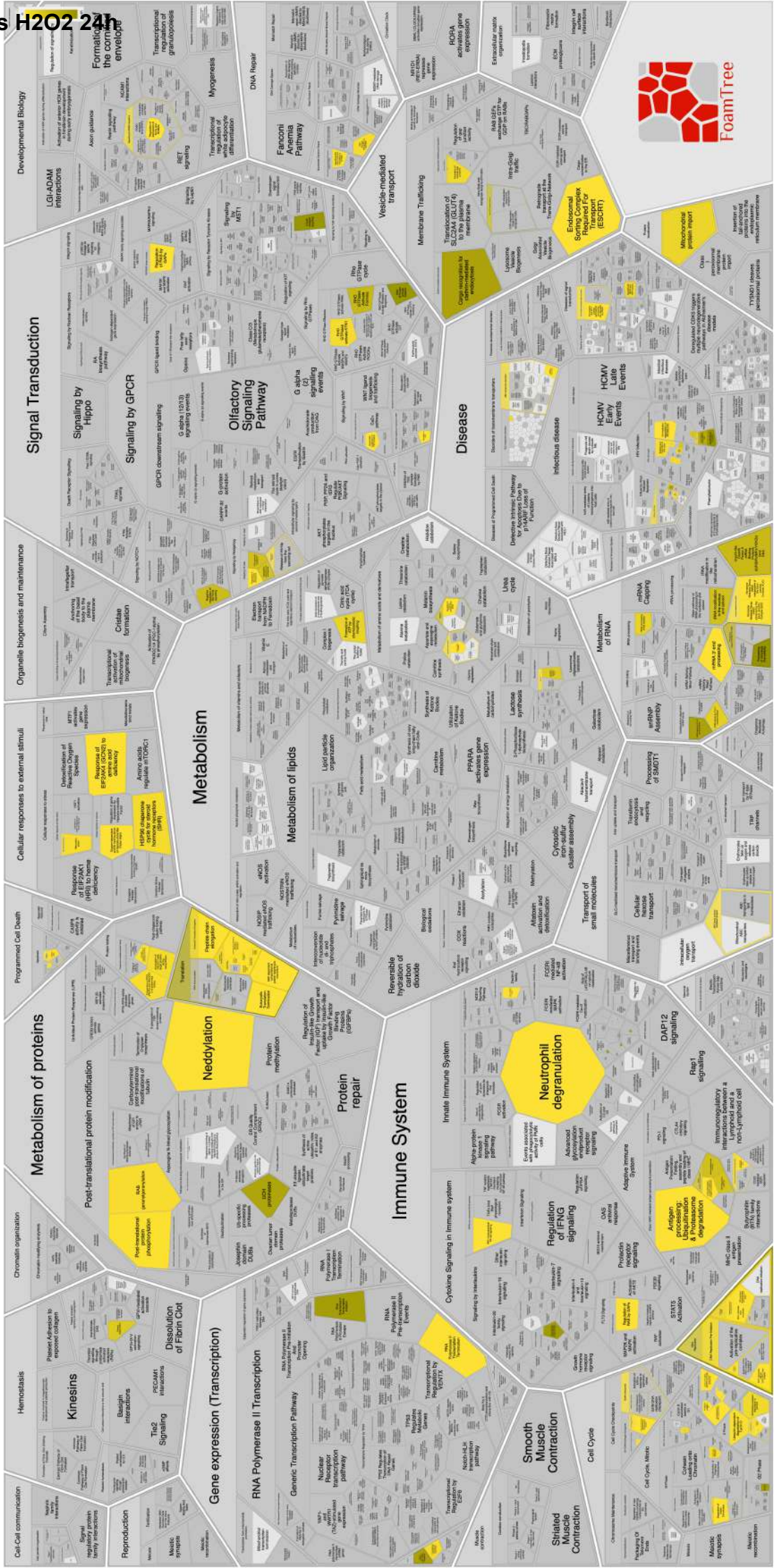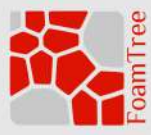

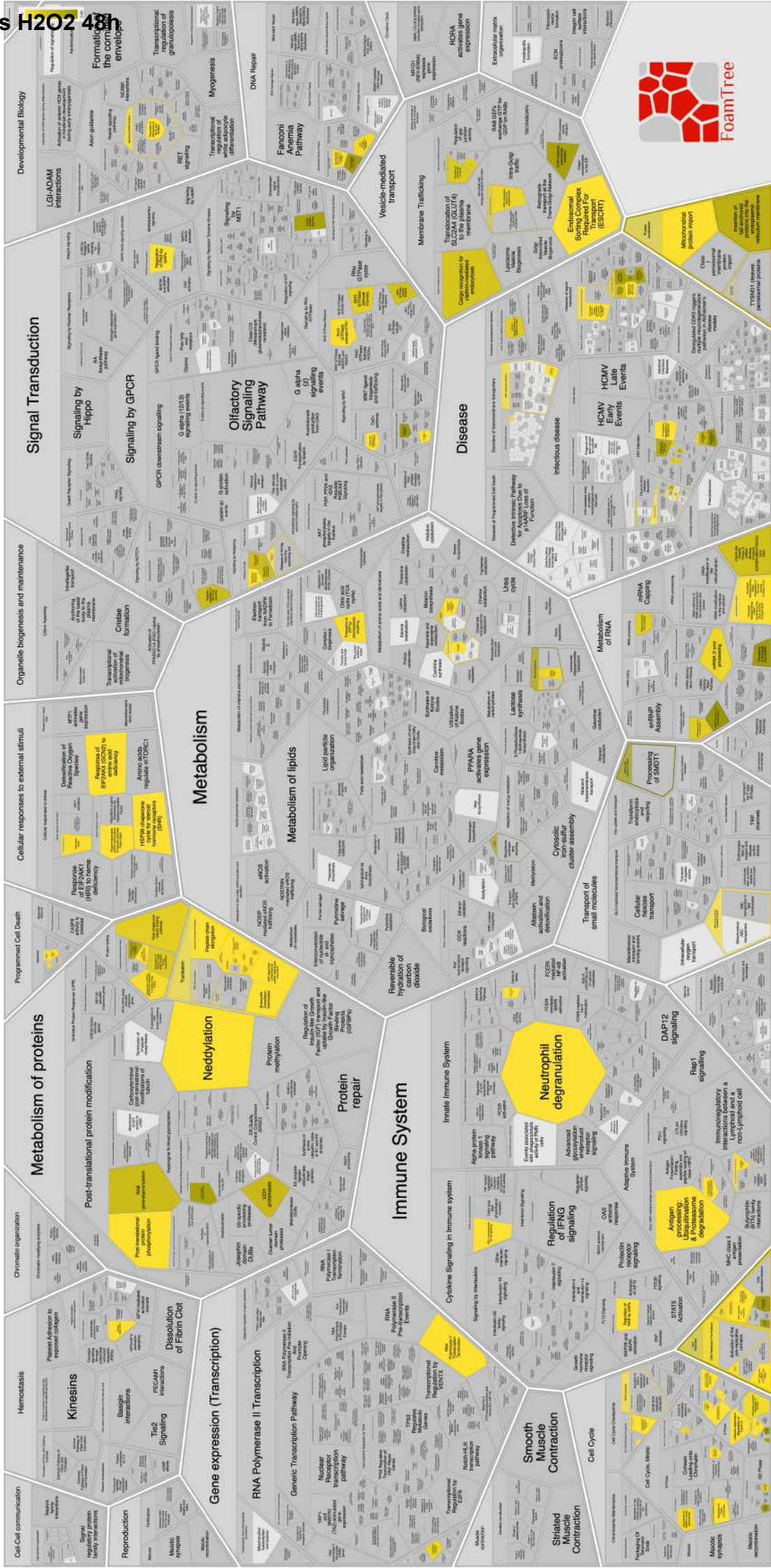

Supplement: Supplementary file 1 [file ijms-22-02064-s001.zip › Supplementary file 8.pdf]
